# Resveratrol Prevents Cytoarchitectural and Interneuronal Alterations in the Valproic Acid Rat Model of Autism

**DOI:** 10.3390/ijms23084075

**Published:** 2022-04-07

**Authors:** Júlio Santos-Terra, Iohanna Deckmann, Giovanna Carello-Collar, Gustavo Della-Flora Nunes, Guilherme Bauer-Negrini, Gustavo Brum Schwingel, Mellanie Fontes-Dutra, Rudimar Riesgo, Carmem Gottfried

**Affiliations:** 1 Translational Research Group in Autism Spectrum Disorder—GETTEA, Universidade Federal do Rio Grande do Sul (UFRGS), Porto Alegre 90040-060, Brazil; juliosterra@gmail.com (J.S.-T.); iohanna.deckmann@gmail.com (I.D.); giovannacollar@gmail.com (G.C.-C.); gustavodfnunes@gmail.com (G.D.-F.N.); negrini.guilherme@gmail.com (G.B.-N.); brumschwingel@gmail.com (G.B.S.); dutra.mellanie@gmail.com (M.F.-D.); rriesgo@hcpa.edu.br (R.R.); 2 Department of Biochemistry, Universidade Federal do Rio Grande do Sul (UFRGS), Porto Alegre 90040-060, Brazil; 3 National Institute of Science and Technology in Neuroimmunomodulation—INCT-NIM, Rio de Janeiro 21040-900, Brazil; 4 Autism Wellbeing and Research Development—AWARD—Initiative BR-UK-CA, Porto Alegre 90040-060, Brazil; 5 Child Neurology Unit, Department of Pediatrics, Hospital de Clínicas de Porto Alegre, Porto Alegre 90035-903, Brazil

**Keywords:** autism spectrum disorder, valproic acid, resveratrol, interneuron, synapse, GABA receptor

## Abstract

Autism spectrum disorder (ASD) is a prevalent neurodevelopmental disorder characterized by several alterations, including disorganized brain cytoarchitecture and excitatory/inhibitory (E/I) imbalance. We aimed to analyze aspects associated with the inhibitory components in ASD, using bioinformatics to develop notions about embryonic life and tissue analysis for postnatal life. We analyzed microarray and RNAseq datasets of embryos from different ASD models, demonstrating that regions involved in neuronal development are affected. We evaluated the effect of prenatal treatment with resveratrol (RSV) on the neuronal organization and quantity of parvalbumin-positive (PV+), somatostatin-positive (SOM+), and calbindin-positive (CB+) GABAergic interneurons, besides the levels of synaptic proteins and GABA receptors in the medial prefrontal cortex (mPFC) and hippocampus (HC) of the ASD model induced by valproic acid (VPA). VPA increased the total number of neurons in the mPFC, while it reduced the number of SOM+ neurons, as well as the proportion of SOM+, PV+, and CB+ neurons (subregion-specific manner), with preventive effects of RSV. In summary, metabolic alterations or gene expression impairments could be induced by VPA, leading to extensive damage in the late developmental stages. By contrast, due to its antioxidant, neuroprotective, and opposite action on histone properties, RSV may avoid damages induced by VPA.

## 1. Introduction

Autism spectrum disorder (ASD) is a neurodevelopmental disorder described as a behavioral dyad composed of (a) communication and social interaction impairments and (b) stereotyped or repetitive patterns of behavior [1]. Besides that, ASD presents a high prevalence (1:44 in children up to 8 years old in the USA) [2] and heterogeneity among individuals [3], resulting in a challenge for clinical diagnosis [4,5] and public health policies [6].

Epilepsy and other electrophysiological abnormalities are among the most prevalent ASD comorbidities, affecting up to 1/3 of the individuals with ASD [7,8,9]. This evidence leads to the most consolidated hypothesis regarding ASD pathophysiology—that the imbalance between excitation and inhibition is probably associated with impairments in the inhibitory component [10,11,12]. Interneurons are crucial for the inhibition of neural circuits [13]. Although they represent only 10–15% of the total neurons in the hippocampus (HC) [14] and 20–30% in the neocortex in humans [15], the high diversity of cellular shapes, populations, and functional properties highlight their importance in the brain [16,17]. Parvalbumin-positive (PV+) and somatostatin-positive (SOM+) interneurons comprise the majority of the GABAergic interneurons (40% and 30% in the cortex, respectively), followed by several smaller populations, including calbindin-positive (CB+) interneurons [16,18]. While SOM+ neurons contribute to the regulation of the local excitatory input integration in cortical regions [19], PV+ neurons are implicated in the integration among different regions [20] and between the hemispheres [21]. Moreover, changes in PV+ neuron inputs [22,23] and the intrinsic features of this subpopulation [24,25] are observed in animal models of ASD, while evidence regarding SOM+ is still incipient. In addition, dysfunctions in other inhibitory components of the E/I balance have already been described in ASD, such as decreased levels of GABA receptor subunits in the parietal cortex and cerebellum in postmortem analysis [26] and synaptic alterations (e.g., reduced pruning [27] and mutations in the genes of PSD-95, gephyrin, and neuroligins [28,29]).

Recently, neuroimmune aspects have emerged as important factors involved in triggering neurodevelopmental disorders. For example, maternal immune activation (MIA) induces ASD-like features, changes in the cytokine profile (especially IL-6), and imbalances in lymphocyte populations [30,31]. In addition, it is observed to alter the expression of genes associated with neurodevelopment, such as genes involved with migration, function, and placement of interneurons [32,33,34]. Similarly, prenatal exposure to valproic acid (VPA) in rodents, a well-established model of autism [35,36,37,38,39], induces interneuronal alterations in sensory areas [36] and HC [40,41]. Furthermore, those animals show alterations in the profile of brain and peripheral cytokines [42] and a reduction of T CD4 + lymphocytes in the lymph nodes [43], indicating a possible involvement of the neuroimmune axis in the VPA model.

Therefore, molecules that prenatally modulate the immune system may hold promise in preventing neurodevelopmental alterations. For example, MR-39, an agonist of the FRP2 receptor, modulates the expression of lipoxin A4 in hippocampal tissues of BTBR and VPA animals, also improving social behavior impairments [44]. Following this line, trans-resveratrol (RSV, 3,5,4’-trihydroxystilbene) has been studied in the context of schizophrenia [45], attention deficit hyperactivity disorder [46], and ASD due to its antioxidant, anti-inflammatory, and neuroprotective effects [47]. The mechanisms associated with the neuroprotective effects of polyphenols, in general, involve scavenging of reactive species of oxygen (and others), modulation of inflammatory cytokines, reduction of the aggregation of amyloid proteins, among several other effects [48]. Interestingly, prenatal treatment with RSV prevented behavioral and molecular impairments in the VPA model [35,36,38]. However, it remains unknown whether RSV exerts any preventive effect on the quantity of GABAergic interneurons and on the laminar organization in the cortex and HC. Thus, we aimed to evaluate RNA-Seq and microarray library datasets in order to identify altered biological pathways in the embryos from an ASD animal model. Subsequently, we aimed to verify which of these pathways could be modulated by RSV; another further goal of this study was to investigate the possible preventive effects of RSV in the VPA model related to GABAergic interneuron proportion and placement; synaptic proteins, and GABA receptor expression in the medial prefrontal cortex (mPFC) and HC from juvenile rats.

## 2. Results

### 2.1. Big Data Evaluation: Early Metabolic Alterations, Cell Cycle Dysfunctions, and Progressive Impairments in Embryos or Progenitor Cells from ASD-Associated Animal Models

In order to create insights regarding cortical embryonic alterations in the VPA model, we refined five datasets (DS) library repositories (Figure S2). The descriptions of the DS are summarized in Table 1.

These analyses helped conduct the evaluation of the experimental results, enabling the creation of more grounded hypotheses about the changes identified in postnatal life. In DS2, we observed an enrichment of differentially expressed genes (DEGs) for pathways associated with carbohydrate metabolism, hypoxia response, and sensory organ development six hours after MIA induction at E12.5 (besides other expected alterations like sensory organ development represented by the eye). Interestingly, 1.83% of these DEGs had an ortholog described in the SFARI database. At E14.5, the lipid, purine, and mitochondrial metabolism were associated with upregulated genes, while the protein dynamics (including histone modification and ubiquitination), cell cycle, nucleic acid metabolism, and response to reactive oxygen species were associated with downregulated genes. Moreover, the cell adhesion, extracellular matrix, synapse, and GABA/glutamate pathways were associated with upregulated DEGs (5.52% of these genes had an ortholog described in the SFARI database). At E18.5, mitochondrial and purine metabolism were still associated with upregulated genes, together with cell adhesion, extracellular matrix, synapse, glutamate metabolism, MAP/ERK, and adenylyl cyclase/cAMP signaling. Protein dynamics (including histone modification and ubiquitination), cell cycle, and nucleic acid metabolism were still associated with downregulated genes, together with WNT, Notch, and Hippo signaling, GABAergic neuron differentiation, and neuronal migration. Of note, 7.6% of the DEGs had an ortholog described in the Simons Foundation Autism Research Initiative (SFARI) database.

In DS5, at E15, upregulated genes were associated with mitochondrial and nucleic acid metabolism, ubiquitination regulation, and cell cycle. On the other hand, downregulated genes were associated with RNA metabolism, gene expression, histone modifications, GABAergic neurons differentiation, and neuronal migration. Interestingly, 5.15% of the DEGs had an ortholog described in the SFARI database.

In DS4, at E14.5, the DEGs identified in different subregions, including cortical subplate, cortical layers, subventricular zone, ganglionic eminence, and neural cells such as interneurons and radial glia, pointed to enriched pathways associated with mitochondrial metabolism, nucleic acid metabolism, protein dynamics, and cell cycle. Of note, 4.45–9.93% of the DEGs had an ortholog described in the SFARI database, depending on the brain region and cell type. At E18.5, the same regions and cells, and also other cortical layers and oligodendrocytes, presented a higher restricted pattern of alterations, especially in the mitochondrial metabolism and protein translation and metabolism. Around 2.91–7.47% of the DEGs had an ortholog described in the SFARI database, except for one region (ganglionic eminence) and one cell (radial glia), which did not present any match with SFARI.

In DS1, the neural cells exposed to VPA demonstrated, after six hours, DEGs associated with nucleic acid metabolism, cell cycle, MAP/ERK, and adenylyl cyclase/cAMP signaling, and neuronal migration, with 5.84% of the DEGs presenting an ortholog described in the SFARI database. After four days, the DEGs were associated with the same pathways, and WNT and Notch signaling, extracellular matrix, cell adhesion, and response to hypoxia. Interestingly, 7.42% of the DEGs had an ortholog described in the SFARI database.

Finally, in DS3, the organoids exposed to VPA demonstrated upregulation of genes associated with carbohydrate and lipid metabolism, ion transport, and cell adhesion. The downregulated genes were associated with nucleic acid and protein metabolism, eye development, synapse, and the WNT pathway. Only 6.36% of the DEGs had an ortholog described in the SFARI database.

### 2.2. The RSV Treatment Prevented the Neuronal Number Alterations Induced by VPA in the mPFC

**Figure 1:**
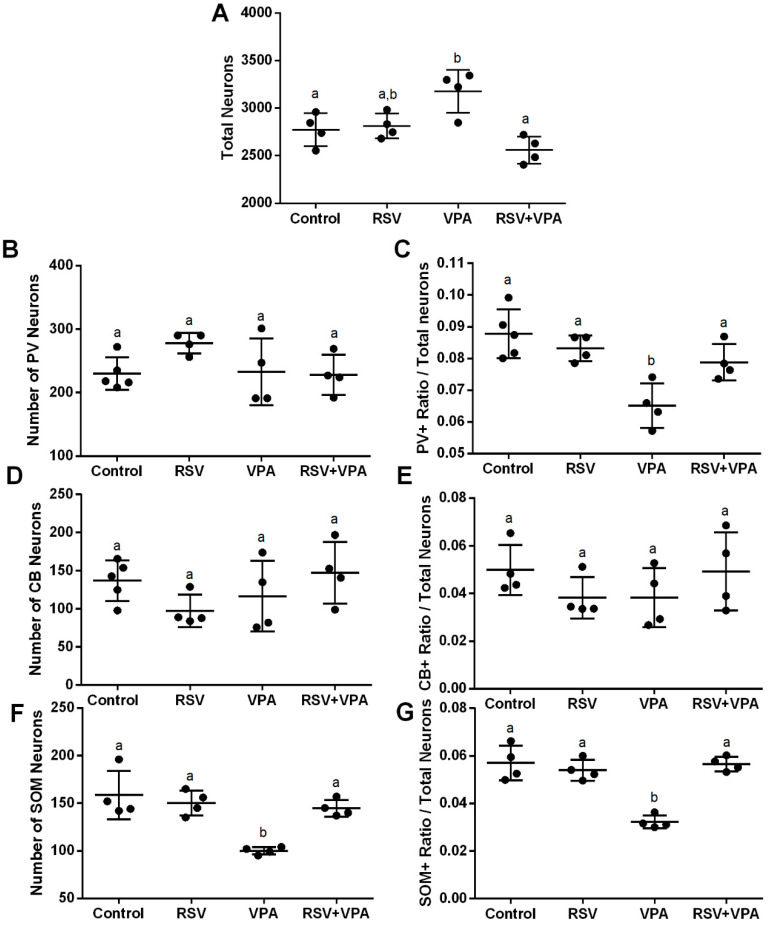
RSV prevents the increase in the number of total neurons, the reduction in the PV+ ratio, and the reduction in the number and ratio of SOM+ induced by VPA in the whole mPFC. (**A**) Quantification of total neurons. (**B**) Quantification of CB+ interneurons. (**C**) Quantification of ratio of CB+ interneurons/total neurons. (**D**) Quantification of PV+ interneurons. (**E**) Quantification of ratio of PV+ interneurons/total neurons (**F**) Quantification of SOM+ interneurons. (**G**) Quantification of the ratio of SOM+ interneurons/total neurons. Values are shown as mean ± standard deviation. Statistical analysis: two-way ANOVA followed by Bonferroni, *p* < 0.05 was considered significant. NCON: 5, NRSV: 4, NVPA: 4, NRSV + VPA:4 CB+NeuN+DAPI, and PV+NeuN+DAPI; N_CON_: 4, N_RSV_: 4, N_VPA_: 4, N_RSV + VPA_:4 and SOM+NeuN+DAPI. Different letters indicate significant differences in the post-test when interaction was significant (*p* < 0.05).

The absolute numbers of total neurons (NeuN + DAPI) and interneurons (CB+ NeuN + DAPI, PV+ NeuN + DAPI, and SOM+ NeuN + DAPI) were counted in each area. The ratio between the number of each interneuron and total neurons is a measurement of the proportion between the inhibitory (interneuron) and excitatory components (the majority of the total neurons). This is done in each subarea of the mPFC and in the mPFC as a whole. The RSV was able to prevent the increased number of total neurons induced by VPA (Figure 1A, interaction factor: F (1, 12) = 14.56, *p =* 0.0025; Cont-VPA ppost-hoc = 0.0361; RSV-VPA ppost-hoc = 0.0627; RSV + VPA-VPA = 0.0016); the decreased ratio of PV+ interneurons (Figure 1E, interaction factor: F (1, 13) = 9.314, *p =* 0.0093; Cont-VPA ppost-hoc = 0.006; RSV-VPA ppost-hoc = 0.0065; RSV + VPA-VPA = 0.0436); and the decreased number of SOM+ interneurons (Figure 1F, interaction factor: F (1, 12) = 12.39, *p =* 0.0042; Cont-VPA ppost-hoc = 0.0008; RSV-VPA ppost-hoc = 0.0030; RSV + VPA-VPA = 0.0074) as well as the SOM+ ratio (Figure 1G, interaction factor: F (1, 12) = 33.09, *p* < 0.0001; Cont-VPA ppost-hoc < 0.0001; RSV-VPA ppost-hoc = 0.0002; RSV + VPA-VPA < 0.0001). PV+ number (Figure 1B), CB+ number (Figure 1D) and CB+ ratio (Figure 1E) did not present significant differences among groups.

### 2.3. The RSV Treatment Prevented the Increased Total Number of Neurons in the Deeper Layers and Whole PrL and IL

The data in Table 2 show that RSV was able to prevent the VPA-induced total neuron increase in deeper layers of PrL (Pre-Limbic Cortex), in the whole PrL, in deeper layers of IL (Infra Limbic Cortex), and in the whole IL. In the upper layers of PrL, a difference between the VPA and VPA-RSV groups was observed. In the deeper layers of aCC (anterior cingulate cortex) and whole aCC, RSV decreased the number of neurons. In the upper layers of IL and aCC, no significant differences were found.

### 2.4. The VPA Induced Alterations in PV+ Number and Ratio in Different Layers of the aCC and PrL

**Figure 2:**
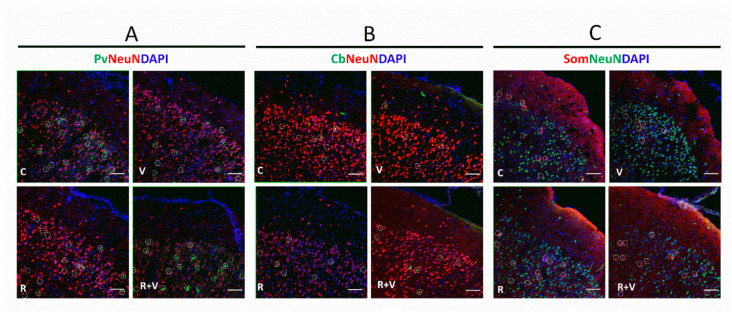
Representative immunofluorescence images of total neurons, PV+, CB+, and SOM+ in the mPFC. Representative images of the aCC, upper layers (II/III). (**A**) Pv, parvalbumin (green); NeuN (red); DAPI (blue). (**B**) Cb, calbindin (green); NeuN (red); DAPI (blue). (**C**) Som, somatostatin (red); NeuN (green); DAPI (blue). Scale bar: 50 µm. The respective interneurons are highlighted within white circles. aCC, anterior cingulate cortex; CB, calbindin-neurons; mPFC, medial frontal cortex; PV, parvalbumin-neurons; SOM, somatostatin-neurons.

The data in Table 3 show that VPA decreased the number of PV+ neurons in the superficial layers of aCC, without RSV prevention. Interestingly, RSV prevented the VPA-induced decrease in PV+ ratio observed in the superficial layers of aCC. The PV+ ratio in the deeper layers of aCC was decreased by VPA, with partial prevention by RSV. When observing the whole aCC, the VPA decreased the PV+ ratio, which was prevented by RSV. In the superficial layers of PrL, the RSV prevented the VPA-induced increase in PV+ number. Regarding the ratio, a tendency was found in the interaction, and differences were identified in the isolated factors. In all the other regions, no differences were found among groups. Illustrative images of PV+ neurons are presented in Figure 2A.

### 2.5. The VPA Induced Alterations in CB+ Ratio in the Upper Layers of aCC, PrL, and IL

The data in Table 4 show that RSV prevented the decreased CB+ ratio induced by VPA in the superficial layers of aCC, but not in the superficial layers of PrL and IL. The differences found in the CB+ number in the superficial layers of aCC, ratio in the whole aCC, ratio in the whole PrL, and ratio in the IL were not associated with any specific factor after the post-hoc test. Illustrative images of PV+ neurons are presented in Figure 2B.

### 2.6. The RSV Prevented the Widespread Impairments Induced by VPA in SOM+ Neurons

The data in Table 5 show that RSV prevented the VPA-induced decrease in SOM+ number and ratio in the superficial layers of aCC. The RSV was also able to prevent the decreased SOM+ number and ratio induced by VPA in the deeper layers of aCC. These results reflected a preventive effect of RSV in the whole aCC in both decreased SOM+ number and ratio. The RSV was also able to prevent the VPA-induced SOM+ ratio decrease in the superficial layers of PrL. In the deeper layers of PrL, RSV + VPA did not differ from any other group, but RSV prevented the reduction in the SOM+ ratio. In the whole PrL, RSV + VPA did not differ from any other group for the SOM+ number, but a prevention was observed in the ratio. In the superficial layers of IL, VPA decreased the numbers of SOM+ neurons. Regarding the ratio, a tendency was found in the interaction, and relevant differences were identified in the isolated factors. In the deeper layers of IL, RSV prevented the VPA-induced decrease of SOM+ neurons in both number and ratio. In the whole IL, RSV prevented the reductions in the SOM+ number and ratio. Illustrative images of PV+ neurons are presented in Figure 2C.

### 2.7. Both VPA and RSV Changed the Levels of Synaptic Proteins, whereas the Level of GABA_A_ Was Affected Only by VPA

**Figure 3:**
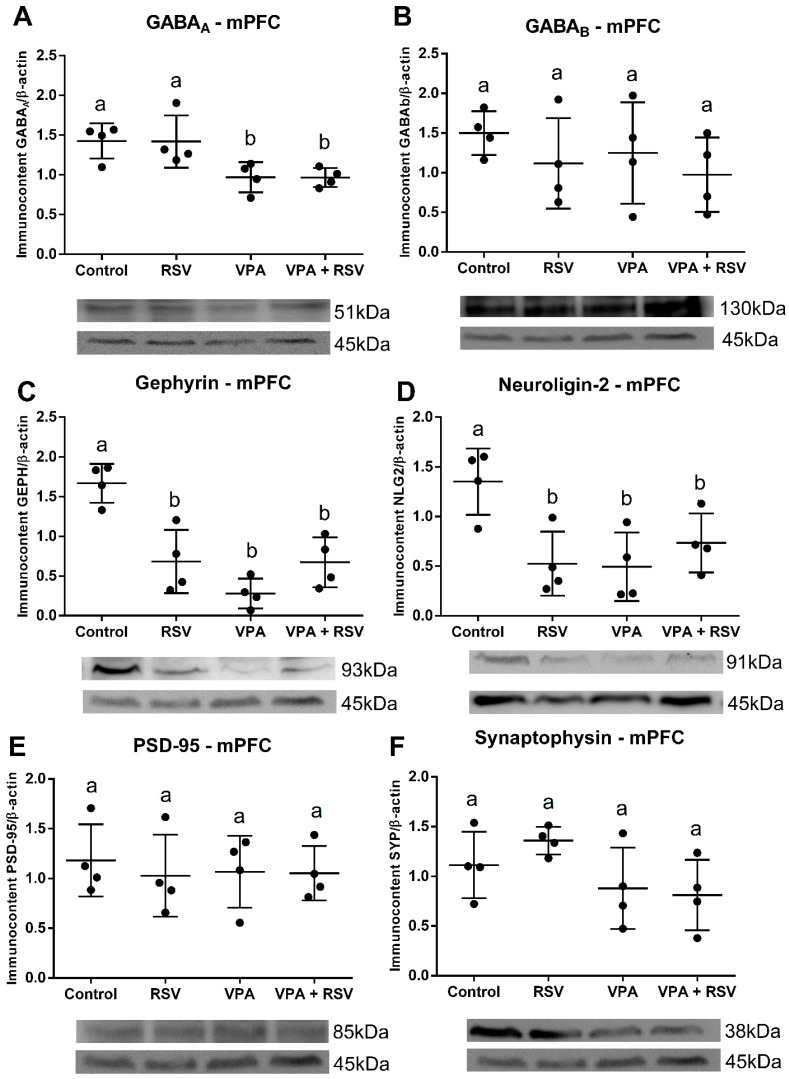
VPA induced a reduction in the immunocontent of GABAA, gephyrin, and neuroligin-2 (the last two also reduced by RSV) in the mPFC. The immunocontent of GABA receptors and synaptic proteins was normalized by the β-actin loading control. Values are shown as mean ± standard deviation. (**A**) GABA_A_ immunocontent. (**B**) GABA_B_ immunocontent. (**C**) Gephyrin immunocontent. (**D**) Neuroligin-2 immunocontent. (**E**) PSD-95 immunocontent. (**F**) Synaptophysin immunocontent. Statistical analysis: two-way ANOVA followed by Bonferroni, *p* < 0.05 was considered significant. N_CON_: 4, N_RSV_: 4, N_VPA_: 4, N_RSV + VPA_:4. Different letters indicate significant differences in the post-test when interaction was significant (*p* < 0.05).

The protein quantification shows that VPA decreased GABA_A_, without RSV prevention (VPA: F (1, 12) = 16.00, *p =* 0.0018) (Figure 3A). Both RSV and VPA decreased gephyrin (Interaction: F (1, 12) = 21.56, *p =* 0.006; Cont-RSV ppost-hoc = 0.0031; Cont-VPAppost-hoc = 0.0001; Cont-RSV + VPAppost-hoc = 0.0029) (Figure 3C) and neuroligin-2 (Interaction: F (1, 12) = 10.77, *p =* 0.0066; Cont-RSV ppost-hoc = 0.0220, Cont-VPAppost-hoc = 0.0172; Cont-RSV + VPA ppost-hoc = 0.1128) (Figure 3D). No differences were observed among groups for GABA_B_ (Figure 3B), PSD-95 (Figure 3E), and synaptophysin (Figure 3F).

### 2.8. The VPA Decreased the Number of Total Neurons and Altered the Ratio of Interneurons in the DG, without Full Prevention by RSV

**Figure 4:**
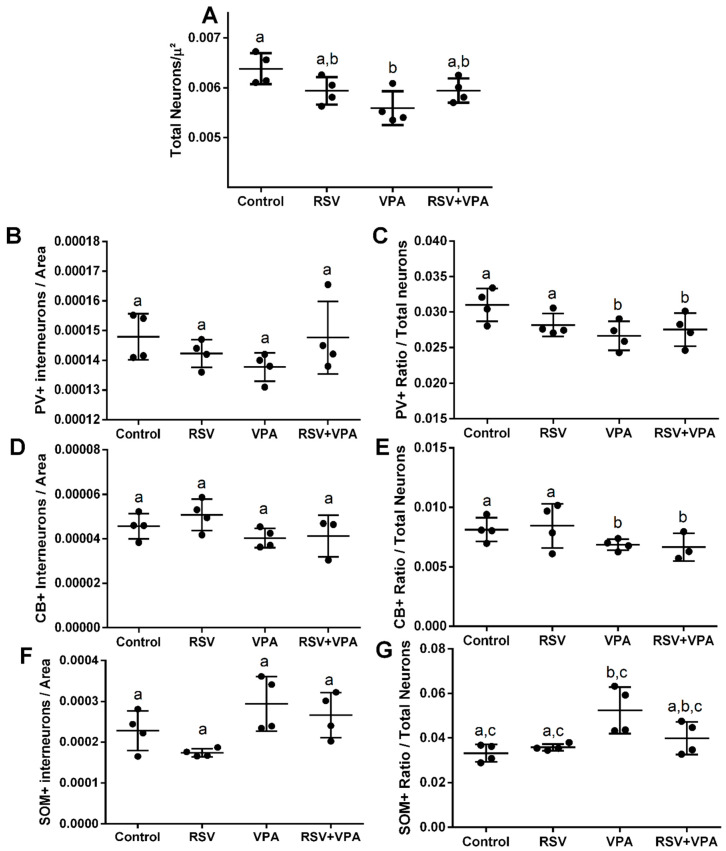
VPA reduced total neurons, CB+ ratio, and PV+ ratio while increasing the SOM+ ratio in the DG without prevention by RSV. (**A**) Quantification of total neurons. (**B**) Quantification of CB+ interneurons. (**C**) Quantification of the ratio of CB+ interneurons/total neurons. (**D**) Quantification of PV+ interneurons. (**E**) Quantification of the ratio of PV+ interneurons/total neurons. (**F**) Quantification of SOM+ interneurons. (**G**) Quantification of the ratio of SOM+ interneurons/total neurons. Values are shown as mean ± standard deviation. Statistical analysis: two-way ANOVA followed by Bonferroni, *p* < 0.05 was considered significant. N_CON_: 4, N_RSV_: 4, N_VPA_: 4, N_RSV+VPA_:4 CB+NeuN+DAPI, and PV+NeuN+DAPI; N_CON_: 4, N_RSV_: 4, N_VPA_: 4, N_RSV+VPA_:3 and SOM+NeuN+DAPI. Different letters indicate significant differences in the post-test when interaction was significant (*p* < 0.05).

**Figure 5:**
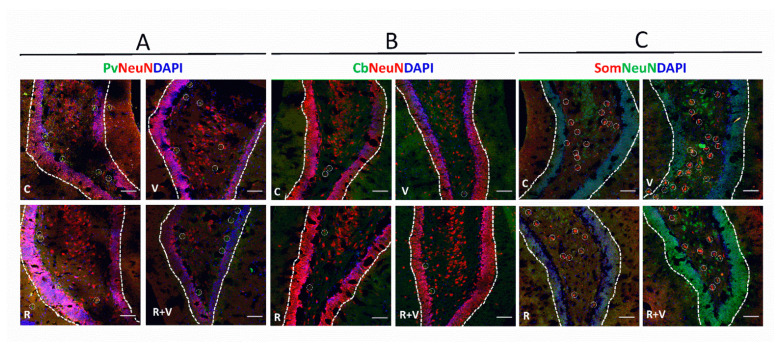
Representative image of total neurons, PV+, CB+, and SOM+ immunofluorescence in the HC. Representative images of the SG, upper layers (II/III). (**A**) Pv, parvalbumin (green); NeuN (red); DAPI (blue). (**B**) Cb, calbindin (green); NeuN (red); DAPI (blue). (**C**) Som, somatostatin (red); NeuN (green); DAPI (blue). Scale bar: 50 µm. The respective interneurons are highlighted within white circles. aCC, anterior cingulate cortex; CB, calbindin-neurons; DG, dentate gyrus; HC, hippocampus; PV, parvalbumin-neurons; SOM, somatostatin-neurons.

The VPA decreased the number of total neurons in DG (Interaction: F (1, 12) = 7.441, *p =* 0.0183; Cont-VPAppost-hoc = 0.0166) (Figure 4A), in PV+ ratio (VPA: F (1, 12) = 5.732, *p =* 0.0339) (Figure 4C), and in CB+ ratio (VPA: F (1, 11) = 5.709, *p =* 0.0359) (Figure 4E), and increased the SOM+ ratio (Interaction: F (1, 12) = 4.840, *p =* 0.0481; Cont-VPA ppost-hoc = 0.0023; RSV-VPA ppost-hoc= 0.0024; RSV + VPA-VPA ppost-hoc = 0.054) (Figure 4G). No differences were observed in the number of PV+ (Figure 4B), CB+ (Figure 4D), and SOM+ neurons (Figure 4F) among groups. Illustrative images of total neurons, PV+, CB+, and SOM+ are presented in Figure 5A–C.

### 2.9. The VPA Altered the Interneuronal Composition in CA1, CA2, CA3, and RSV Presented a per se Effect in CA3

The VPA group decreased the CB+ number in CA1, CA2, and CA3, following a decreased ratio of these neurons in CA2 and CA3 (Supplementary Materials Table S3). The RSV had a per se effect in CA3, decreasing PV+ numbers, but not altering the ratio (Supplementary Materials Table S2). The VPA increased the SOM+ number and ratio in the CA2, while RSV had a per se effect in CA3, increasing the number of SOM+ neurons without affecting the ratio (Supplementary Materials Table S4). For the total neurons, a significant difference was only found between VPA and RSV + VPA groups in CA2 (Supplementary Materials Table S1). No differences were found in other parameters.

### 2.10. The Immunocontent of the Analyzed Proteins Did Not Differ among Groups in the Hippocampus

**Figure 6:**
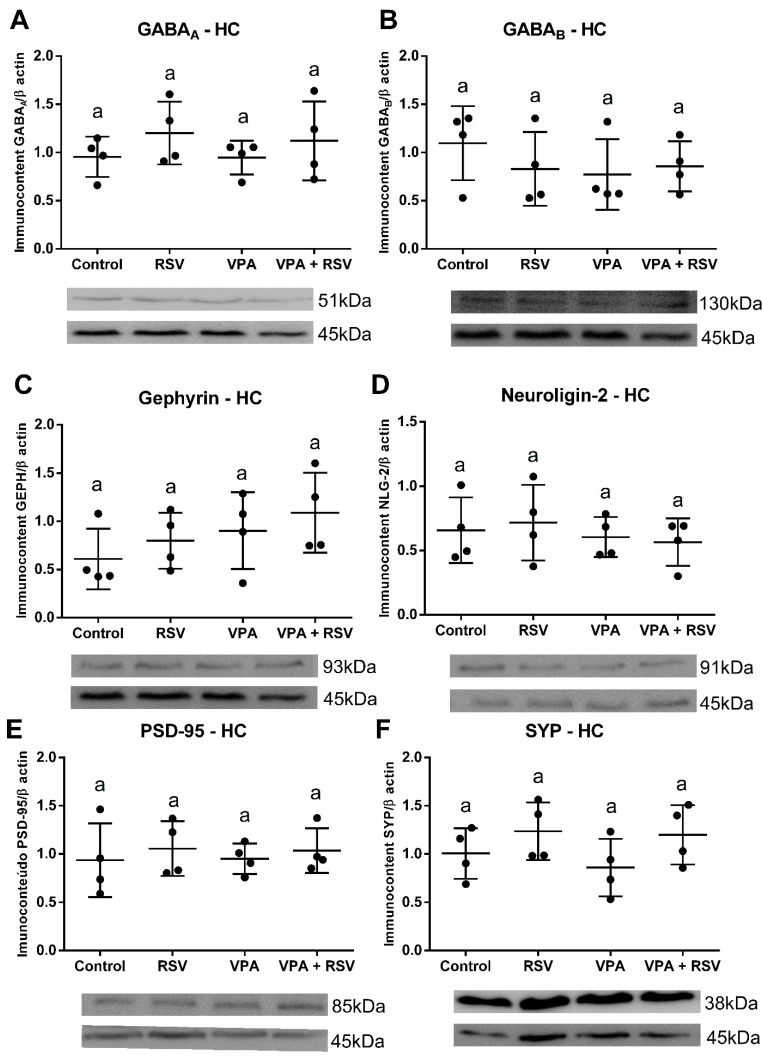
There were no significant differences in the expression of synaptic proteins and GABA receptors in the HC. The immunocontent of GABA receptors and synaptic proteins was normalized by the β-actin loading control. Values are shown as mean ± standard deviation. (**A**) GABA_A_ immunocontent. (**B**) GABA_B_ immunocontent. (**C**) Gephyrin immunocontent. (**D**) Neuroligin-2 immunocontent. (**E**) PSD-95 immunocontent. (**F**) Synaptophysin immunocontent. Statistical analysis: two-way ANOVA followed by Bonferroni, *p* < 0.05 was considered significant. N_CON_: 4, N_RSV_: 4, N_VPA_: 4, N_RSV + VPA_:4.

In the HC, no significant differences were found for all parameters evaluated. GABA_A_ (Interaction: F (1, 12) = 0.06436 *p =* 0.8040. VPA: F (1, 12) = 0.09175 p= 0.7672. RSV: F (1, 12) = 2.034 *p* = 0.1793) (Figure 6A), GABA_B_ (Interaction: F (1, 12) = 0.9989 *p =* 0.3373. VPA: F (1, 12) = 0.7182 *p =* 0.4133. RSV: F (1, 12) = 0.2652 *p =* 0.6159) (Figure 6B), gephyrin (Interaction: F (1, 12) = 8.221 × 10^−5^ *p =* 0.9929. VPA: F (1, 12) = 2.657 *p =* 0.1291. RSV: F (1, 12) = 1.099 *p =* 0.3152) (Figure 6C), neuroligin-2 (Interaction: F (1, 12) = 0.1844 *p =* 0.6753. VPA: F (1, 12) = 0.8125 *p =* 0.3851. RSV: F (1, 12) = 0.007832 *p =* 0.9309) (Figure 6D), PSD-95 (Interaction: F (1, 12) = 0.01751 *p =* 0.8969. VPA: F (1, 12) = 0.001065 *p =* 0.9745. RSV: F (1, 12) = 0.5443 *p =* 0.4748) (Figure 6E), and synaptophysin (Interaction: F (1, 12) = 0.1378 *p =* 0.7169. VPA: F (1, 12) = 0.3949 *p =* 0.5415. RSV: F (1, 12) = 3.797 *p =* 0.0751) (Figure 6F).

## 3. Discussion

Changes in the organization of brain cytoarchitecture directly impact not only the local circuits but also the integration among different brain regions. In the autistic brain, cortical disorganization [49,50] and both high local connectivity and low long-range connectivity have already been described [51]. Here, we first studied microarray/RNA-Seq repository datasets of embryos from ASD animal models in order to investigate enriched pathways for the DEGs identified in them.

Firstly, the carbohydrate metabolic imbalance observed in E12.5 in DS2 was also observed in the organoids exposed to VPA (DS3), indicating that this may be the starting point of several subsequent alterations. The proliferation of neuronal progenitors relies mostly on aerobic glycolysis as the energetic source [52]; thus, an alteration in this metabolic pathway may induce early proliferative issues. In the subsequent days (E14.5 and E17.5), the pathways appear to induce a general condition of acceleration of neuronal differentiation, with upregulation of adhesion, neurotransmitter, and synaptic pathways to the detriment of the cell-cycle, gene expression, and protein dynamics regulation. Many of these features are also observed in brain organoids exposed to VPA (DS3), probably impacting the final disposition and organization of the neurons in different brain regions.

VPA has already demonstrated an influence in carbohydrate metabolism and mitochondrial function [53], increasing the production of reactive oxygen species [54]. RSV is a known antioxidant and anti-inflammatory molecule, and, thus, the early treatment (starting in E6.5) may attenuate a possible metabolic alteration induced by VPA. Moreover, VPA is a known inhibitor of histone deacetylases [55,56], while RSV is an activator of sirtuins [57], which may counteract the alterations in gene expression and cell-cycle. Thus, RSV may create a neuroprotective background, preventing alterations caused by VPA and expansion of initial damage throughout embryonic life, resulting in the maintenance of the neuronal composition in the mPFC and HC (to a lesser extent) in postnatal life.

Considering these data and our previous data from adult animals of the VPA animal model (P120) [41], which presented alterations in the neuronal composition of the HC, including disturbances in PV+, CB+, and SOM+, we studied here the same structure and also expanded it for mPFC in young animals (P30). Now, we demonstrate a substantial disorganization in the mPFC and HC neuronal cytoarchitecture in the VPA group, as well as important preventive effects of prenatal treatment with RSV, especially in the mPFC.

The VPA group showed an increased number of total neurons, while the interneurons presented either a reduced ratio or number in the mPFC, depending on the subpopulation. This numerical increase (even not significant in some subregions) is relevant because the ratio of interneurons/total neurons can be influenced by subtle alterations. We demonstrated that the deeper layers of PrL and IL presented the most significant increase in the number of total neurons. Postmortem analysis of ASD patients already demonstrated an increased number of neurons in the mini-columns of the frontal and parietal cortex [49] and patches of disorganization in the cortical layers, especially in the deeper layers [50]; moreover, an increase in the number of total neurons was observed in the dorsolateral cortex, the homologous region to the mPFC [58] in humans.

Next, we explored the gene expression datasets to identify potential mechanisms that could underlie the increased number of neurons in the cortex of VPA mice. The majority of the cortical neurons are excitatory pyramidal cells (about 75–80%) [59]. These neurons are generated in the ventricular zone in the early stages of embryogenesis (around E10 in rodents) [60] and reach the cortex through radial migration from the cortical subplate. VPA animals display an increased number of non-GABAergic neurons and thickness of the cortical layers, concomitant with changes in the expression of cell cycle proteins, suggesting maintenance of the proliferative phase for a longer time [61]. When we observed DS4 gene expression data, we noticed that the cortical subplate, the migrating cells from the subventricular zone, and even the radial glia (directly associated with migration guidance) displayed DEGs associated with cell cycle and gene expression in E14.

Next, we investigated the distribution of specific interneuron subpopulations in the mPFC. Prenatal exposure to VPA reduced the number of GABAergic SOM+ interneurons and the proportion of SOM+ and PV+ interneurons, with no general effect on CB+ (only specific alterations in the subregions). PV+, SOM+, and CB+ are mostly generated in different segments of the GE, developing a migration route that starts around E12.5 in rodents (the same day as the prenatal exposure to VPA) [18].

The SOM+ neurons originate in the medial portion of the GE (MGE) through an initial signaling system based on the increase in SHH expression followed by the expression of the NKX2.1 factor [62]. Since this interneuron population was the only one whose absolute number changed and considering that their migration starts earlier, it is possible that the drastic damage induced by VPA may occur when these cells are still in the proliferative stages. Interestingly, prenatal exposure to VPA at E9.5 reduced the SHH expression in E11.5 embryos [63], which could explain the SOM+ impairments.

On the other hand, the absence of changes in the absolute number (already described in the mPFC of the VPA model [64]) along with the reduction in the ratio of PV+ may suggest a subtle change potentially associated with migration processes, as seen in the anomalous pattern of distribution throughout the subregions. Indeed, while the aCC showed a reduction in number and proportion, the upper layers of PrL showed a completely opposite pattern. Previously, we observed that VPA animals showed an increased proportion of PV+ neurons in the upper layers of the somatosensory area, which was prevented by RSV [36]. CB+ presented a similar pattern to PV+ in relation to the ratio, and these subtle alterations may be associated with the small percentage of this interneuron population.

Interestingly, in DS4, the GE and emerging interneurons at E14 presented major alterations in cell-cycle, gene expression, and protein dynamics, which could result in alterations in the interneuronal proliferation and migration since they are strictly regulated by a sequence of transcription factors, including SHH, NKX2.1, DLX, LHX, SOX.

In addition to the changes in the number and proportion of GABAergic neurons, prenatal exposure to VPA induced a reduction in the immunocontent of the GABAA receptor, a finding already observed in postmortem analysis of ASD patients in the aCC [65] and in the frontal and parietal cortices [26]. Moreover, this alteration possibly contributes to the histological changes observed because this receptor plays an important role in neuronal migration throughout development [66]. Finally, the similar effect of VPA and RSV in reducing the immunocontent of gephyrin and neuroligin-2, two major constituents of inhibitory synapses, may point to an involvement of the Notch pathway, a signaling route highlighted in the DS2 as an altered pathway in late embryonic life, which involved the modulation of synapses [67,68] and is capable of being modulated by both VPA and RSV [69]. However, RSV alone did not cause major histological or behavioral alterations, similar to what was shown in previous studies from our research group [35,36,38].

In the HC, it was possible to observe that prenatal exposure to VPA mainly induced alterations in the DG. The reduction in the total neurons and the alterations in the interneurons, especially SOM+, may induce circuit imbalances with other regions, especially the mPFC, given the important role of SOM+ in integrating the HC and mPFC [70]. VPA is known to reduce neurogenesis in the HC [71] and induce the misplacement of neurons through a pathway mediated by the CXCL12 chemokine and its receptor, CXCR4 [72], which also plays a role in the migration of interneurons. Alterations in CB+ are present in several regions; however, the relatively low abundance of these cells in the HC may hinder accurate quantification. RSV has already demonstrated effects on the modulation of HC interneurons in adults [73]. Thus, prenatal treatment with RSV may cause alterations in the fate of these cells in specific situations. The absence of alterations in the synaptic proteins and GABA receptors in the HC suggests that VPA effects in this region may be restricted to modulation of neuronal populations and organization of brain cytoarchitecture.

## 4. Materials and Methods

### 4.1. Animals

Wistar rats from the Center for Reproduction and Experimentation of Laboratory Animals (CREAL) were housed in the bioterium of the Department of Biochemistry at UFRGS and maintained under a standard 12/12 h light/dark cycle at a constant temperature of 22 ± 2 °C with food and water ad libitum. The Ethics Commission of the Federal University of Rio Grande do Sul approved this project (CEUA-UFRGS #35733). The animals were euthanized by an anesthetic overdose of ketamine (300 mg/kg) and xylazine (40 mg/kg) (concentrations three times higher than the concentration required to obtain an anesthetic-surgical plan). All experimental procedures were performed in accordance with ethical principles in accordance with the Euthanasia Practice Guidelines of the National Council for Animal Experimentation Control (CONCEA) (Normative Resolution N. 13, 2013), NIH Guide for the Care and Use of Laboratory Animals, as well as Brazilian Arouca Law (11,794 of 8 October 2008).

### 4.2. Drugs and Prenatal Treatments

Wistar rats were mated overnight, and pregnancy was confirmed the next morning through the presence of spermatozoa in the female’s vaginal smear; when the pregnancy was confirmed, that day was considered the embryonic day 0.5 (E0.5). From E6.5 to E18.5, the pregnant rats received a daily subcutaneous injection of RSV (Fluxome, Stenløse, Denmark) at 3.6 mg/kg or equivalent volume of vehicle (dimethyl sulfoxide P.A. (DMSO)), as previously described [35,36]. At E12.5, pregnant rats received a single intraperitoneal injection of either VPA at 600 mg/kg (Acros Organics, Morris Plains, Morris County, NJ, USA) or vehicle (saline solution 0.9%). The four experimental groups, according to the treatment received, were the following: Control (vehicles), RSV, VPA, and RSV + VPA. Pregnant rats were singly housed at E18 for parturition. We considered the day of birth to be postnatal day 0 (P0). The female pups were euthanized at postnatal day (P) P21, and only males were used in this work. After weaning at P21, the male offspring were kept until P30. The total number of animals used in the study was nine control, eight RSV, eight VPA, and eight RSV + VPA divided randomly in experiments, generated from five control dams, four RSV, nine VPA, and nine RSV + VPA (the excedent offspring was destined to other projects, ensuring full use of the biological material). Loss rate for the VPA groups was approximately 50% in this protocol. The sample size used in each experiment is described in the corresponding figures and/or tables.

### 4.3. Immunofluorescence

The tissues were fixed and cryopreserved in OCT^®^ and cut in a Leica^®^ cryostat (−20 °C). The slices (25 µm) corresponding to the mPFC and HC were placed on histological slides covered with poly-L-lysine and post-fixed with 4% paraformaldehyde. The brain coordinates were the following: bregma 3.72/3.24 (mPFC and subregions: anterior cingulate [aCC], prelimbic [PrL], and infralimbic [IL]) and −2.92/−3.00 (HC and subregions: dentate gyrus [DG], CA1, CA2, and CA3) according to Paxinos Atlas (5th edition) [74]. Three slices were alternately placed in each histological slide, stained with specific primary antibodies for NeuN combined with PV, SOM, or CB, in addition to corresponding secondary antibodies associated with a fluorophore and nuclear DAPI dye according to the protocol described by Fontes-Dutra et al. [36]. Technical information and concentrations of the reagents used in the immunofluorescence assays are summarized in Supplementary Materials Table S1. The images were obtained using the Olympus FV1000^®^ confocal microscope at the Center for Microscopy and Microanalysis (CMM-UFRGS) (Supplementary Figure S1 demonstrates the subdivisions established for the analyzed regions). Each coronal section was photographed in stacks by the confocal microscope (8, on average; dimensions: 635.9 × 635.9 microns). The analyses were performed manually by two trained researchers who were blinded to the experimental groups using the Cell Counter plug-in in the ImageJ^®^ software [75]. Quantification was conducted by counting the cells in 8 stacks of at least 2 slices per animal (all stacks were counted individually and with the overlapping image).

The results are shown as the absolute number of total neurons (NeuN+DAPI) and interneurons (CB+NeuN+DAPI, PV+NeuN+DAPI, and SOM+NeuN+DAPI) normalized by area and as the ratio between the number of interneurons and total neurons to obtain a proportion between the inhibitory (interneuron) and excitatory components (the majority of the total neurons) according to the following formula: (CB+, PV+ or SOM+) Interneurons/Total neurons (based on Fontes-Dutra et al. [36]. This ratio was made separately for each interneuron evaluated. The mPFC was subdivided into three subregions, named aCC, PrL, and IL. Each of these regions were subdivided into upper layers (II/III) and deeper layers (IV/V). The total number of neurons, the number of each interneuron (PV+, CB+, and SOM+), and the ratio (interneuron/total neurons) were evaluated in each subfield (i.e., upper layers of aCC, deeper layers of aCC, upper layers of PrL, deeper layers of PrL, upper layers of IL, deeper layers of IL). The amount observed in the deeper + upper layers of a subregion represents the whole subregion (i.e., deeper layers of aCC + upper layers of aCC = whole aCC). The amount observed in whole aCC + whole PrL + whole IL represents the whole mPFC. The HC was subdivided into four subregions: DG, CA1, CA2, and CA3. In each of them, the total number of neurons, the number of each interneuron (PV+, CB+, and SOM+), and the ratio (interneuron/total neurons) were evaluated.

### 4.4. Western Blotting

Samples from mPFC and HC were homogenized and prepared in a buffer containing 10% SDS, 100 mM EDTA, 500 mM TRIS/HCl buffer (pH 8), and protease inhibitors. The supernatant was collected after centrifugation at 14.000× *g* for 20 min at 4 °C. Total proteins were quantified by the Lowry method [76], and the samples were prepared in a buffer containing glycerol, bromophenol blue, 500 mM TRIS/HCl buffer, and β-mercaptoethanol. Equal amounts of protein (40 µg) were applied to 10% polyacrylamide gels, separated by unidimensional electrophoresis, and transferred to nitrocellulose membranes to detect the immunocontent of GABA_A_, GABA_B_, gephyrin, neuroligin-2, PSD-95 and synaptophysin proteins using specific primary antibodies according to the protocol adapted from Deckmann et al., [77]. Technical information and concentrations of the reagents used in the Western Blotting assays are summarized in Supplementary Materials Table S1. After incubation with corresponding secondary peroxidase-associated antibodies (HRP), the chemiluminescent signal was detected using the ImageQuant™ LAS 4000 system (GE HealthCare Life Sciences^®^, Chicago, IL, USA). The quantification of the relative protein content was performed with the ImageJ^®^ software, and the data were normalized by the endogenous marker β-actin.

### 4.5. Transcriptomic Analysis

To provide insights into the embryonic processes that could lead to the alterations observed in the postnatal brain of ASD models, we selected five RNA-Seq and microarray datasets [32,78,79,80,81] from MIA animal models, VPA-exposed cell cultures, and cortical organoids (Table 1) since databases of VPA-induced animal models are not available yet. The differentially expressed genes (DEGs) of each dataset were analyzed with Cytoscape^®^ [82] using the BiNGO^®^ plug-in [83] to evaluate Gene Ontology (GO) enrichments in a determined set of genes, providing tables with the statistically significant most representative GO terms. We also compared the DEGs observed in each dataset with the Simons Foundation Autism Research Initiative (SFARI) gene database [84] to observe the percentage of DEGs that have an ortholog already described as altered in ASD.

### 4.6. Statistical Analysis

All the analyses were performed using the GraphPad Prism 6 software (GraphPad Software, La Jolla, CA, USA). Kolmogorov–Smirnov and Shapiro–Wilk tests of normality were applied to determine the data distribution. As the data presented a normal distribution, we chose a parametric test (two-way ANOVA) followed by a Bonferroni post-test. When there was an interaction effect, pairwise comparison was analyzed in the post-hoc; when there was no effect, the effect of exposure to factors (VPA or RSV) was analyzed.

## 5. Conclusions

The present study demonstrated that the prenatal treatment with RSV was able to prevent important alterations in the neuronal composition of the mPFC induced by prenatal exposure to VPA, probably improving parameters associated with the E/I balance. These findings are in accordance with several other studies that have already demonstrated the neuroprotective effects of RSV in psychiatric disorders, not only in animal models but also in humans, highlighting the translational value of the study. The transcriptomic analysis allowed the establishment of hypotheses to explain the developmental context of these interventions, highlighting the pathways such as WNT, NOTCH, and others in which VPA and RSV may act. Next, we demonstrated that prenatal exposure to VPA alters the neuronal profile in the mPFC and HC, impacting the number and proportion of interneurons, indicating a possible E/I imbalance. Moreover, VPA also induced alterations in the immunocontent of a GABA receptor and synaptic proteins in the mPFC, adding another layer of evidence to comprehend the alterations in the circuitry of this region. In summary, prenatal treatment with RSV was able to prevent neuronal alterations in the mPFC. In addition, our analyses suggest that the investigation of mechanisms involved in the development of interneurons, brain cytoarchitecture, and synaptic content can be a promising strategy to expand the understanding of the pathophysiology of ASD.

## Figures and Tables

**Table 1:** Description of the datasets analyzed.

Reference	Animal Model/Sample	Embryonic Day	Method
Balmer et al., 2014 (DS1)	Neural differentiated hESC exposed to VPA	6 h and 4 days after VPA exposure	Microarray
Canales et al., 2021 (DS2)	MIA Poly(I:C), mouse cortex	E12.5, E14.5, and E17.5	RNA-Seq
Cui et al., 2020 (DS3)	Cortical organoids exposed to VPA	5 days after exposure	RNA-Seq
Kalish et al., 2021 (DS4)	MIA Poly(I:C), mouse brain	E14 and E18	RNA-Seq (single-cell)
Oskvig et al., 2012 (DS5)	MIA Poly(I:C), rat cortex	E15	Microarray

**Table 2:** Distribution profile of total neurons in the mPFC.

	Mean ± SD	F (DFn. DFd); *p*-Value	Pairwise Comparisons	
Total Neurons aCC (II/III)	CON: 336.00 ± 39.75 RSV: 321.00 ± 42.35 VPA: 371.25 ± 59.35 RSV + VPA: 318.75 ± 41.60	Interaction: F (1, 12) = 0.6523 *p =* 0.4350 VPA: F (1, 12) = 0.5052 *p =* 0.4908 RSV: F (1, 12) = 2.113 *p =* 0.1717	CON vs. RSV:	>0.9999
			CON vs. VPA:	>0.9999
			CON vs. RSV + VPA:	>0.9999
			RSV vs. VPA:	0.9108
			RSV vs. RSV + VPA:	>0.9999
			VPA vs. RSV + VPA:	0.8147
Total Neurons aCC (IV/V)	CON: 555.25 ± 76.01 RSV: 513.00 ± 13.71 VPA: 650.25 ± 49.85 RSV + VPA: 506.75 ± 39.78	Interaction: F (1, 12) = 4.086 *p =* 0.0661 # VPA: F (1, 12) = 3.140 *p =* 0.1018 RSV: F (1, 12) = 13.75 *p =* 0.0030 **	CON vs. RSV:	>0.9999
			CON vs. VPA:	0.1197
			CON vs. RSV + VPA:	>0.9999
			RSV vs. VPA:	0.0132 *
			RSV vs. RSV + VPA:	>0.9999
			VPA vs. RSV + VPA:	0.0096 **
Total Neurons Whole aCC	CON: 891.25 ± 104.45 RSV: 834.00 ± 51,153 VPA: 1021.00 ± 107.97 RSV + VPA: 825.50 ± 71.11	Interaction: F (1, 12) = 2.546 *p =* 0.1365 VPA: F (1, 12) = 1.960 *p =* 0.1868 RSV: F (1, 12) = 8.482 *p =* 0.0130 *	CON vs. RSV:	>0.9999
			CON vs. VPA:	0.3342
			CON vs. RSV + VPA:	>0.9999
			RSV vs. VPA:	0.0606 #
			RSV vs. RSV + VPA:	>0.9999
			VPA vs. RSV + VPA:	0.0469 *
Total Neurons PrL (II/III)	CON: 368.25 ± 55.12 RSV: 363.50 ± 14.91 VPA: 404.75 ± 28.15 RSV + VPA: 325.75 ± 13.25	Interaction: F (1, 12) = 5.214 *p =* 0.0414 * VPA: F (1, 12) = 0.001478 *p =* 0.9700 RSV: F (1, 12) = 6.633 *p =* 0.0243 *	CON vs. RSV:	>0.9999
			CON vs. VPA:	0.8305
			CON vs. RSV + VPA:	0.5360
			RSV vs. VPA:	0.5882
			RSV vs. RSV + VPA:	0.7594
			VPA vs. RSV + VPA:	0.0296 *
Total Neurons PrL (IV/V)	CON: 572.50 ± 22.10 RSV: 611.25 ± 62.50 VPA: 693.00 ± 49.22 RSV + VPA: 536.00 ± 49.43	Interaction: F (1, 12) = 16.54 *p =* 0.0016 ** VPA: F (1, 12) = 0.8838 *p =* 0.3657 RSV: F (1, 12) = 6.036 *p =* 0.0302 *	CON vs. RSV:	>0.9999
			CON vs. VPA:	0.0244 *
			CON vs. RSV + VPA:	>0.9999
			RSV vs. VPA:	0.2004
			RSV vs. RSV + VPA:	0.2832
			VPA vs. RSV + VPA:	0.0036 **
Total Neurons Whole PrL	CON: 940.75 ± 63.10 RSV: 974.75 ± 76.61 VPA: 1097.75 ± 61.32 RSV + VPA: 861.75 ± 60.10	Interaction: F (1, 12) = 16.93 *p =* 0.0014 ** F (1, 12) = 0.4497 *p =* 0.5152 F (1, 12) = 9.478 *p =* 0.0096 **	CON vs. RSV:	>0.9999
			CON vs. VPA:	0.0326 *
			CON vs. RSV + VPA:	0.6861
			RSV vs. VPA:	>0.1268
			RSV vs. RSV + VPA:	0.1884
			VPA vs. RSV + VPA:	0.0016 **
Total Neurons IL (II/III)	CON: 348.75 ± 47.98 RSV: 355.75 ± 26.98 VPA: 363.25 ± 20.85 RSV + VPA: 315.25 ± 17.41	Interaction: F (1, 12) =3.49 *p =* 0.0880 # VPA: F (1, 12) = 0.7707 *p =* 0.3972 RSV: F (1, 12) = 1.917 p =0.1914	CON vs. RSV:	>0.9999
			CON vs. VPA:	>0.9999
			CON vs. RSV + VPA:	0.8139
			RSV vs. VPA:	>0.9999
			RSV vs. RSV + VPA:	0.4623
			VPA vs. RSV + VPA:	0.2447
Total Neurons IL (IV/V)	CON: 593.75 ± 39.22 RSV: 646.00 ± 26.24 VPA: 696.50 ± 51.39 RSV + VPA: 557.00 ± 54.16	Interaction: F (1, 12) = 18.75 *p =* 0.0010 ** VPA: F (1, 12) = 0.1041 *p =* 0.7525 RSV: F (1, 12) = 3.858 *p =* 0.0731 #	CON vs. RSV:	0.7209
			CON vs. VPA:	0.0387 *
			CON vs. RSV + VPA:	>0.9999
			RSV vs. VPA:	0.7910
			RSV vs. RSV + VPA:	0.0904 #
			VPA vs. RSV + VPA:	0.0047 **
Total Neurons Whole IL	CON: 942.50 ± 27.47 RSV: 1001.75 ± 35.08 VPA: 1059.75 ± 66.74 RSV + VPA: 872.75 ± 53.72	Interaction: F (1, 12) = 26.01 *p =* 0.0003 *** VPA: F (1, 12) = 0.05921 *p =* 0.8119 RSV: F (1, 12) = 7.000 *p =* 0.0213	CON vs. RSV:	0.6496
			CON vs. VPA:	0.0297 *
			CON vs. RSV + VPA:	0.3820
			RSV vs. VPA:	0.6908
			RSV vs. RSV + VPA:	0.0158 *
			VPA vs. RSV + VPA:	0.0008 ***

II/III, upper cortical layers; IV/V, deeper cortical layers; aCC, anterior cingulate cortex; IL, infralimbic cortex; mPFC, medial prefrontal cortex; PrL, prelimbic cortex; SD, standard deviation. *p* < 0.05 considered significant. * *p* < 0.05. ** *p* < 0.01, *** *p* < 0.001, # trend. Statistical analyses: two-way ANOVA parametric test followed by Bonferroni. N_CON_: 4. N_RSV_: 4. N_VPA_: 4. N_RSV + VPA_: 4.

**Table 3:** Distribution profile of PV neurons in the mPFC.

	Mean ± SD	F (DFn. DFd); *p*-Value	Pairwise Comparisons	
PV Total aCC (II/III)	CON: 30.200 ± 2.863564 RSV: 28.750 ± 5.560276 VPA: 21.250 ± 7.274384 RSV + VPA: 25.000 ± 0.816	Interaction: F (1, 13) = 1.292 *p =* 0.2761 VPA: F (1, 13) = 7.709 *p =* 0.0157 * RSV: F (1, 13) = 0.2528 *p =* 0.6235	CON vs. RSV:	>0.9999
			CON vs. VPA:	0.0830
			CON vs. RSV + VPA:	0.7351
			RSV vs. VPA:	0.2497
			RSV vs. RSV + VPA:	>0.9999
			VPA vs. RSV + VPA:	>0.9999
PV Ratio aCC (II/III)	CON: 0.0804 ± 0.005 RSV: 0.0812 ± 0.017 VPA: 0.0506 ± 0.009 RSV + VPA: 0.0777 ± 0.005	Interaction: F (1, 13) = 6.862 *p =* 0.0212 * VPA: F (1, 13) = 11.06 *p =* 0.0055 ** RSV: F (1, 13) = 7.772 *p =* 0.0154 *	CON vs. RSV:	0.9999
			CON vs. VPA:	0.0050 **
			CON vs. RSV + VPA:	0.9999
			RSV vs. VPA:	0.0061 **
			RSV vs. RSV + VPA:	0.9999
			VPA vs. RSV + VPA:	0.0152 *
PV Total aCC (IV/V)	CON: 51.800 ± 8.55 RSV: 50.500 ± 13.89 VPA: 36.750 ± 15.37 RSV + VPA: 49.500 ± 10.96	Interaction: F (1, 13) = 1.391 *p =* 0.2593 VPA: F (1, 13) = 1.816 *p =* 0.2009 RSV: F (1, 13) = 0.9240 *p =* 0.3540	CON vs. RSV:	0.9999
			CON vs. VPA:	0.5361
			CON vs. RSV + VPA:	0.9999
			RSV vs. VPA:	0.8135
			RSV vs. RSV + VPA:	0.9999
			VPA vs. RSV + VPA:	0.9834
PV Ratio aCC (IV/V)	CON: 0.107 ± 0.0145 RSV: 0.0856 ± 0.01586 VPA: 0.0548 ± 0.0168 RSV + VPA: 0.0840 ± 0.019	Interaction: F (1, 13) = 9.992 *p =* 0.0075 ** VPA: F (1, 13) = 11.16 *p =* 0.0053 ** RSV: F (1, 13) = 0.2128 *p =* 0.6522	CON vs. RSV:	0.4297
			CON vs. VPA:	0.0024
			CON vs. RSV + VPA:	0.3398
			RSV vs. VPA:	0.1270
			RSV vs. RSV + VPA:	>0.9999
			VPA vs. RSV + VPA:	0.1606
PV Total Whole aCC	CON: 82.000 ± 11.25 RSV: 78.500 ± 16.60 VPA: 59.750 ± 23.60 RSV + VPA:74.500 ± 10.87	Interaction: F (1, 13) = 1.354 *p =* 0.2655 VPA: F (1, 13) = 2.801 *p =* 0.1181 RSV: F (1, 13) = 0.5144 *p =* 0.4859	CON vs. RSV:	0.9999
			CON vs. VPA:	0.3593
			CON vs. RSV + VPA:	0.9999
			RSV vs. VPA:	0.7401
			RSV vs. RSV + VPA:	0.9999
			VPA vs. RSV + VPA:	0.9999
PV Ratio Whole aCC	CON: 0.094 ± 0.009 RSV: 0.084 ± 0.014 VPA: 0.053 ± 0.012 RSV + VPA 0.081 ± 0.011	Interaction: F (1, 13) = 11.45 *p =* 0.0049 ** VPA: F (1, 13) = 14.87 *p =* 0.0020 ** RSV: F (1, 13) = 2.065 *p =* 0.1744	CON vs. RSV:	0.9999
			CON vs. VPA:	0.0009 ***
			CON vs. RSV + VPA:	0.6141
			RSV vs. VPA:	0.0177 *
			RSV vs. RSV + VPA:	0.9999
			VPA vs. RSV + VPA:	0.0330 *
PV Total PrL (II/III)	CON: 28.400 ± 2.88 RSV:24.500 ± 7.04 VPA: 38.750 ± 3.77 RSV + VPA:26.250 ± 6.94	Interaction: F (1, 13) = 4.159 *p =* 0.0483 * VPA: F (1, 13) = 7.359 *p =* 0.0178 * RSV: F (1, 13) = 7.675 *p =* 0.0159 *	CON vs. RSV:	0.9999
			CON vs. VPA:	0.0257 *
			CON vs. RSV + VPA:	0.9999
			RSV vs. VPA:	0.0138 *
			RSV vs. RSV + VPA:	0.9999
			VPA vs. RSV + VPA:	0.0335 *
PV Ratio PrL (II/III)	CON: 0.072 ± 0.004 RSV: 0.066 ± 0.009 VPA: 0.087 ± 0.003 RSV + VPA 0.070 ± 0.007	Interaction: F (1, 13) = 2.679 *p =* 0.0930 # VPA: F (1, 13) = 7.172 *p =* 0.0190 * RSV: F (1, 13) = 15.25 *p =* 0.0018 **	CON vs. RSV:	0.7397
			CON vs. VPA:	0.0474 *
			CON vs. RSV + VPA:	0.9999
			RSV vs. VPA:	0.0033 **
			RSV vs. RSV + VPA:	0.9999
			VPA vs. RSV + VPA:	0.0128 *
PV Total PrL (IV/V)	CON: 49.200 ± 7.82 RSV: 56.000 ± 6.83 VPA: 50.750 ± 5.12 RSV + VPA: 48.750 ± 12.57	Interaction: F (1, 13) = 1.129 *p =* 0.3072 VPA: F (1, 13) = 0.4738 *p =* 0.5033 RSV: F (1, 13) = 0.3360 *p =* 0.5720	CON vs. RSV:	>0.9999
			CON vs. VPA:	>0.9999
			CON vs. RSV + VPA:	>0.9999
			RSV vs. VPA:	>0.9999
			RSV vs. RSV + VPA:	>0.9999
			VPA vs. RSV + VPA:	>0.9999
PV Ratio PrL (IV/V)	CON: 0.096 ± 0.020 RSV:0.084 ± 0.009 VPA: 0.068 ± 0.010 RSV + VPA:0.082 ± 0.020	Interaction: F (1, 13) = 2.460 *p =* 0.1408 VPA: F (1, 13) = 3.640 *p =* 0.0787 # RSV: F (1, 13) = 0.001322 *p =* 0.9715	CON vs. RSV:	0.9999
			CON vs. VPA:	0.1520
			CON vs. RSV + VPA:	0.9999
			RSV vs. VPA:	0.9999
			RSV vs. RSV + VPA:	0.9999
			VPA vs. RSV + VPA:	0.9999
PV Total Whole PrL	CON: 74.400 ± 8.82 RSV: 80.500 ± 13.52 VPA: 86.500 ± 9.000 RSV + VPA: 75.000 ± 17.92	Interaction: F (1, 13) = 2.051 *p =* 0.1758 VPA: F (1, 13) = 0.2884 *p =* 0.6003 RSV: F (1, 13) = 0.1930 *p =* 0.6676	CON vs. RSV:	>0.9999
			CON vs. VPA:	>0.9999
			CON vs. RSV + VPA:	>0.9999
			RSV vs. VPA:	>0.9999
			RSV vs. RSV + VPA:	>0.9999
			VPA vs. RSV + VPA:	>0.9999
PV Ratio Whole PrL	CON: 0.088 ± 0.013 RSV:0.078 ± 0.006 VPA: 0.072 ± 0.001 RSV + VPA:0.077 ± 0.014	Interaction: F (1, 13) = 2.101 *p =* 0.1709 VPA: F (1, 13) = 2.834 *p =* 0.1161 RSV: F (1, 13) = 0.3874 *p =* 0.5444	CON vs. RSV:	0.9372
			CON vs. VPA:	0.2425
			CON vs. RSV + VPA:	0.7067
			RSV vs. VPA:	0.9999
			RSV vs. RSV + VPA:	0.9999
			VPA vs. RSV + VPA:	0.9999
PV Total IL (II/III)	CON: 28.800 ± 5.90 RSV: 29.250 ± 10.25 VPA: 33.750 ± 5.12 RSV + VPA: 25.000 ± 4.69	Interaction: F (1, 13) = 1.936 *p =* 0.1875 VPA: F (1, 13) = 0.01121 *p =* 0.9173 RSV: F (1, 13) = 1.576 *p =* 0.2315	CON vs. RSV:	>0.9999
			CON vs. VPA:	>0.9999
			CON vs. RSV + VPA:	>0.9999
			RSV vs. VPA:	>0.9999
			RSV vs. RSV + VPA:	>0.9999
			VPA vs. RSV + VPA:	0.5473
PV Ratio IL (II/III)	CON: 0.082 ± 0.010 RSV: 0.070 ± 0.015 VPA: 0.070 ± 0.010 RSV + VPA: 0.075 ± 0.008	Interaction: F (1, 13) = 2.644 *p =* 0.1279 VPA: F (1, 13) = 0.4062 *p =* 0.5350 RSV: F (1, 13) = 0.3412 *p =* 0.5691	CON vs. RSV:	0.7940
			CON vs. VPA:	0.7443
			CON vs. RSV + VPA:	>0.9999
			RSV vs. VPA:	>0.9999
			RSV vs. RSV + VPA:	>0.9999
			VPA vs. RSV + VPA:	>0.9999
PV Total IL (IV/V)	CON: 45.40 ± 9.50 RSV: 56.00 ± 28.25 VPA: 48.00 ± 12.355 RSV + VPA: 53.50 ± 11.80	Interaction: F (1, 13) = 0.1646 *p =* 0.6915 VPA: F (1, 13) = 0.001360 *p =* 0.9711 RSV: F (1, 13) = 1.171 *p =* 0.2989	CON vs. RSV:	>0.9999
			CON vs. VPA:	>0.9999
			CON vs. RSV + VPA:	>0.9999
			RSV vs. VPA:	>0.9999
			RSV vs. RSV + VPA:	>0.9999
			VPA vs. RSV + VPA:	>0.9999
PV Ratio IL (IV/V)	CON:0.080 ± 0.020 RSV: 0.073 ± 0.031 VPA: 0.067 ± 0.016 RSV + VPA: 0.081 ± 0.020	Interaction: F (1, 13) = 1.046 *p =* 0.3251 VPA: F (1, 13) = 0.04663 *p =* 0.8324 RSV: F (1, 13) = 0.1055 *p =* 0.7504	CON vs. RSV:	>0.9999
			CON vs. VPA:	>0.9999
			CON vs. RSV + VPA:	>0.9999
			RSV vs. VPA:	>0.9999
			RSV vs. RSV + VPA:	>0.9999
			VPA vs. RSV + VPA:	>0.9999
PV Total Whole IL	CON: 74.60 ± 8.50 RSV: 80.50 ± 13.52 VPA: 81.75 ± 15.25 RSV + VPA: 77.50 ± 15.25	Interaction: F (1, 13) = 0.6249 *p =* 0.4434 VPA: F (1, 13) = 0.1045 *p =* 0.7517 RSV: F (1, 13) = 0.01651 *p =* 0.8997	CON vs. RSV:	>0.9999
			CON vs. VPA:	>0.9999
			CON vs. RSV + VPA:	>0.9999
			RSV vs. VPA:	>0.9999
			RSV vs. RSV + VPA:	>0.9999
			VPA vs. RSV + VPA:	>0.9999
PV Ratio Whole IL	CON: 0.082 ± 0.015 RSV: 0.072 ± 0.024 VPA: 0.068 ± 0.013 RSV + VPA: 0.079 ± 0.013	Interaction: F (1, 13) = 1.533 *p =* 0.2376 VPA: F (1, 13) = 0.1634 *p =* 0.6926 RSV: F (1, 13) = 0.006546 *p =* 0.9367	CON vs. RSV:	>0.9999
			CON vs. VPA:	>0.9999
			CON vs. RSV + VPA:	>0.9999
			RSV vs. VPA:	>0.9999
			RSV vs. RSV + VPA:	>0.9999
			VPA vs. RSV + VPA:	>0.9999

II/III, upper cortical layers; IV/V, deeper cortical layers; aCC, anterior cingulate cortex; IL, infralimbic cortex; mPFC, medial prefrontal cortex; PrL, prelimbic cortex; PV, parvalbumin-positive interneuron; SD, standard deviation. *p* < 0.05 was considered significant. * *p* < 0.05. ** *p* < 0.01, *** *p* < 0.001, # trend. Statistical analyses: two-way ANOVA parametric test followed by Bonferroni. N_CON_: 4. N_RSV_: 4. N_VPA_: 4. N_RSV + VPA_: 4.

**Table 4:** Distribution profile of CB neurons in the mPFC.

	Mean ± SD	F (DFn. DFd); *p*-Value	Pairwise Comparisons	
CB Total aCC (II/III)	CON: 19.20 ± 6.87 RSV: 16.75 ± 2.50 VPA: 13.25 ± 6.70 RSV + VPA: 21.75 ± 3.59	Interaction: F (1, 13) = 6.023 *p =* 0.0290 * VPA: F (1, 13) = 0.03959 *p =* 0.8454 RSV: F (1, 13) = 2.327 *p =* 0.1511	CON vs. RSV:	0.9999
			CON vs. VPA:	0.7519
			CON vs. RSV + VPA:	0.9999
			RSV vs. VPA:	0.9999
			RSV vs. RSV + VPA:	0.5430
			VPA vs. RSV + VPA:	0.1006
CB Ratio aCC (II/III)	CON: 0.057 ± 0.005 RSV: 0.050 ± 0.006 VPA: 0.034 ± 0.010 RSV + VPA: 0.064 ± 0.014	Interaction: F (1, 13) = 18.31 *p =* 0.0009 *** VPA: F (1, 13) = 1.081 *p =* 0.3175 RSV: F (1, 13) = 6.006 *p =* 0.0292	CON vs. RSV:	0.9999
			CON vs. VPA:	0.0117 *
			CON vs. RSV + VPA:	0.9999
			RSV vs. VPA:	0.1905
			RSV vs. RSV + VPA:	0.2626
			VPA vs. RSV + VPA:	0.0028 **
CB Total aCC (IV/V)	CON: 22.00 ± 7.25 RSV: 17.00 ± 4.45 VPA: 22.75 ± 10.00 RSV + VPA: 22.5 ± 9.95	Interaction: F (1, 13) = 0.4352 *p =* 0.5210 VPA: F (1, 13) = 0.7193 *p =* 0.4117 RSV: F (1, 13) = 0.5220 *p =* 0.4828	CON vs. RSV:	0.9999
			CON vs. VPA:	0.9999
			CON vs. RSV + VPA:	0.9999
			RSV vs. VPA:	0.9999
			RSV vs. RSV + VPA:	0.9999
			VPA vs. RSV + VPA:	0.9999
CB Ratio aCC (IV/V)	CON: 0.059 ± 0.024 RSV: 0.037 ± 0.012 VPA: 0.039 ± 0.011 RSV + VPA: 0.042 ± 0.020	Interaction: F (1, 13) = 2.304 *p =* 0.1530 VPA:F (1, 13) = 0.7381 *p =* 0.4058 RSV:F (1, 13) = 1.381 *p =* 0.2610	CON vs. RSV:	0.4334
			CON vs. VPA:	0.6472
			CON vs. RSV + VPA:	0.9794
			RSV vs. VPA:	0.9999
			RSV vs. RSV + VPA:	0.9999
			VPA vs. RSV + VPA:	0.9999
CB Total Whole aCC	CON: 37.2 ± 13.92 RSV: 28.25 ± 8.15 VPA: 38.75 ± 19.77 RSV + VPA: 39.25 ± 17.40	Interaction: F (1, 13) = 0.4000 *p =* 0.5381 VPA: F (1, 13) = 0.7055 *p =* 0.4161 RSV: (1, 13) = 0.3198 *p =* 0.5813	CON vs. RSV:	0.9999
			CON vs. VPA:	0.9999
			CON vs. RSV + VPA:	0.9999
			RSV vs. VPA:	0.9999
			RSV vs. RSV + VPA:	0.9999
			VPA vs. RSV + VPA:	0.9999
CB Ratio Whole aCC	CON: 0.058 ± 0.014 RSV: 0.042 ± 0.009 VPA: 0.038 ± 0.010 RSV + VPA 0.051 ± 0.017	Interaction: F (1, 13) = 5.701 *p =* 0.0328 * VPA: F (1, 13) = 0.9515 *p =* 0.3471 RSV: F (1, 13) = 0.03555 *p =* 0.8534	CON vs. RSV:	0.5037
			CON vs. VPA:	0.1775
			CON vs. RSV + VPA:	0.9999
			RSV vs. VPA:	0.9999
			RSV vs. RSV + VPA:	0.9999
			VPA vs. RSV + VPA:	0.9213
CB Total PrL (II/III)	CON: 26.20 ± 7.35 RSV: 18.25 ± 1.70 VPA: 16.00 ± 9.75 RSV + VPA:19.25 ± 5.45	Interaction: F (1, 13) = 2.859 *p =* 0.1147 VPA: F (1, 13) = 1.929 *p =* 0.1882 RSV: F (1, 13) = 0.5036 *p =* 0.4905	CON vs. RSV:	0.6284
			CON vs. VPA:	0.2603
			CON vs. RSV + VPA:	0.9078
			RSV vs. VPA:	0.9999
			RSV vs. RSV + VPA:	0.9999
			VPA vs. RSV + VPA:	0.9999
CB Ratio PrL (II/III)	CON: 0.079 ± 0.025 RSV: 0.059 ± 0.009 VPA: 0.034 ± 0.015 RSV + VPA: 0.057 ± 0.016	Interaction: F (1, 13) = 6.149 *p =* 0.0276 VPA: F (1, 13) = 7.593 *p =* 0.0164 RSV: F (1, 13) = 0.01238 *p =* 0.9131	CON vs. RSV:	0.6541
			CON vs. VPA:	0.0132
			CON vs. RSV + VPA:	0.4615
			RSV vs. VPA:	0.4187
			RSV vs. RSV + VPA:	0.9999
			VPA vs. RSV + VPA:	0.5849
CB Total PrL (IV/V)	CON: 23.20 ± 4.35 RSV: 19.00 ± 4.95 VPA: 26.75 ± 14.05 RSV + VPA: 33.2 ± 7.65	Interaction: F (1, 13) = 1,748 *p =* 0.2089 VPA: F (1, 13) = 4.837 *p =* 0.0709 # RSV: F (1, 13) = 0.04947 *p =* 0.8275	CON vs. RSV:	0.9999
			CON vs. VPA:	0.9999
			CON vs. RSV + VPA:	0.6120
			RSV vs. VPA:	0.9999
			RSV vs. RSV + VPA:	0.1830
			VPA vs. RSV + VPA:	0.9999
CB Ratio PrL (IV/V)	CON: 0.054 ± 0.018 RSV: 0.034 ± 0.009 VPA: 0.041 ± 0.017 RSV + VPA: 0.050 ± 0.014	Interaction: F (1, 13) = 3.755 *p =* 0.0747 VPA F (1, 13) = 0.04387 *p =* 0.8373 RSV: F (1, 13) = 0.6898 *p =* 0.4212	CON vs. RSV:	0.3931
			CON vs. VPA:	0.9999
			CON vs. RSV + VPA:	0.9999
			RSV vs. VPA:	0.9999
			RSV vs. RSV + VPA:	0.9763
			VPA vs. RSV + VPA:	0.9999
CB Total Whole PrL	CON: 51.00 ± 7.87 RSV: 36.25 ± 6.95 VPA: 42.75 ± 19.25 RSV + VPA:52.25 ± 7.90	interaction: F (1, 12) = 3.243 *p =* 0.0969 VPA: F (1, 12) = 0.3312 *p =* 0.5756 RSV: F (1, 12) = 0.1520 *p =* 0.7035	CON vs. RSV:	0.8841
			CON vs. VPA:	0.9999
			CON vs. RSV + VPA:	0.9999
			RSV vs. VPA:	0.9999
			RSV vs. RSV + VPA:	0.7124
			VPA vs. RSV + VPA:	0.9999
CB Ratio Whole PrL	CON: 0.062 ± 0.016 RSV: 0.043 ± 0.08 VPA: 0.038 ± 0.016 RSV + VPA: 0.052 ± 0.014	Interaction: F (1, 13) = 5.736 *p =* 0.0324 * VPA: F (1, 13) = 0.4439 *p =* 0.5169 RSV: F (1, 13) = 0.2759 *p =* 0.6082	CON vs. RSV:	0.3221
			CON vs. VPA:	0.2670
			CON vs. RSV + VPA:	0.9999
			RSV vs. VPA:	0.9999
			RSV vs. RSV + VPA:	0.9999
			VPA vs. RSV + VPA:	0.9999
CB Total IL (II/III)	CON: 18.75 ± 5.50 RSV: 14.25 ± 4.70 VPA: 14.25 ± 7.00 RSV + VPA: 26.00 ± 11.5	Interaction: F (1, 11) = 3.947 *p =* 0.0662 # VPA: F (1, 11) = 7.953 *p =* 0.4461 RSV: F (1, 11) = 12.79 *p =* 0.4471	CON vs. RSV:	0.9999
			CON vs. VPA:	0.9999
			CON vs. RSV + VPA:	0.9999
			RSV vs. VPA:	0.9999
			RSV vs. RSV + VPA:	0.4421
			VPA vs. RSV + VPA:	0.4421
CB Ratio IL (II/III)	CON: 0.069 ± 0.018 RSV: 0.043 ± 0.013 VPA: 0.031 ± 0.007 RSV + VPA: 0.060 ± 0.023	Interaction: F (1, 13) = 13.42 *p =* 0.0029 ** VPA: F (1, 13) = 1.476 *p =* 0.2460 RSV: F (1, 13) = 0.01159 *p =* 0.9159	CON vs. RSV:	0.1364
			CON vs. VPA:	0.0216 *
			CON vs. RSV + VPA:	0.9999
			RSV vs. VPA:	0.9999
			RSV vs. RSV + VPA:	0.6924
			VPA vs. RSV + VPA:	0.1324
CB Total IL (IV/V)	CON: 29.00 ± 10.90 RSV: 19.00 ± 6.13 VPA: 26.25.00 ± 6.18 RSV + VPA: 30.00 ± 8.25	Interaction: F (1, 13) = 3.990 *p =* 0.0672 # VPA: F (1, 13) = 1,271 *p =* 0.2800 RSV: F (1, 13) = 0.5051 *p =* 0.4898	CON vs. RSV:	0.4251
			CON vs. VPA:	0.9999
			CON vs. RSV + VPA:	0.9999
			RSV vs. VPA:	0.9999
			RSV vs. RSV + VPA:	0.3037
			VPA vs. RSV + VPA:	0.9999
CB Ratio IL (IV/V)	CON: 0.045 ± 0.010 RSV: 0.035 ± 0.013 VPA: 0.040 ± 0.010 RSV + VPA: 0.048 ± 0.014	Interaction: F (1, 12) = 2.805 *p =* 0.1198 VPA: F (1, 12) = 0.3000 *p =* 0.5939 RSV: F (1, 12) = 0.07687 *p =* 0.7863	CON vs. RSV:	0.9999
			CON vs. VPA:	0.9999
			CON vs. RSV + VPA:	0.9999
			RSV vs. VPA:	0.9999
			RSV vs. RSV + VPA:	0.8522
			VPA vs. RSV + VPA:	0.9999
CB Total Whole IL	CON: 44.25 ± 9.45 RSV: 33.00 ± 9.70 VPA: 40.25 ± 12.00 RSV + VPA: 55.00 ± 18.65	Interaction: F (1, 13) = 5.378 *p =* 0.0597 # VPA: F (1, 13) = 0.7738 *p =* 0.3950 RSV: F (1, 13) = 0.01655 *p =* 0.8996	CON vs. RSV:	0.5925
			CON vs. VPA:	0.9999
			CON vs. RSV + VPA:	0.9999
			RSV vs. VPA:	0.9999
			RSV vs. RSV + VPA:	0.2766
			VPA vs. RSV + VPA:	0.9303
CB Ratio Whole IL	CON: 0.64 ± 0.027 RSV: 0.39 ± 0.011 VPA: 0.36 ± 0.008 RSV + VPA: 0.53 ± 0.018	Interaction: F (1, 13) = 5.736 *p =* 0.0324 * VPA: F (1, 13) = 0.4439 *p =* 0.5169 RSV: F (1, 13) = 0.2759 *p =* 0.6082	CON vs. RSV:	0.3221
			CON vs. VPA:	0.2670
			CON vs. RSV + VPA:	0.9999
			RSV vs. VPA:	0.9999
			RSV vs. RSV + VPA:	0.9999
			VPA vs. RSV + VPA:	0.9999

II/III, upper cortical layers; IV/V, deeper cortical layers; aCC, anterior cingulate cortex; CB, calbindin-positive interneuron; IL, infralimbic cortex; mPFC, medial prefrontal cortex; PrL, prelimbic cortex; SD, standard deviation. *p* < 0.05 considered significant. * *p* < 0.05, ** *p* < 0.01, *** *p* < 0.001, # trend. Statistical analyses: two-way ANOVA parametric test followed by Bonferroni. N_CON_: 5. N_RSV_: 4. N_VPA_: 4. N_RSV + VPA_: 4.

**Table 5:** Distribution profile of SOM neurons in the mPFC.

	Mean ± SD	F (DFn. DFd); *p*-Value	Pairwise Comparisons	
SOM Total aCC (II/III)	CON: 18.5 ± 2.65 RSV: 17.5 ± 0.5 VPA: 12 ± 2.50 RSV + VPA: 18.75 ± 2.00	Interaction: F (1, 12) = 13.66 *p =* 0.0031 ** VPA: F (1, 12) = 6.270 *p =* 0.0277 * RSV: F (1, 12) = 7.521 *p =* 0.0178 *	CON vs. RSV:	>0.9999
			CON vs. VPA:	0.0053 **
			CON vs. RSV + VPA:	>0.9999
			RSV vs. VPA:	0.0179 *
			RSV vs. RSV + VPA:	>0.9999
			VPA vs. RSV + VPA:	0.0040 **
SOM Ratio aCC (II/III)	CON: 0.060 ± 0.009 RSV: 0.059 ± 0.008 VPA: 0.034 ± 0.007 RSV + VPA: 0.068 ± 0.002	Interaction: F (1, 12) = 25.14 *p =* 0.0003 *** VPA: F (1, 12) = 6.460 *p =* 0.0259 * RSV: F (1, 12) = 22.93 *p =* 0.0004 ***	CON vs. RSV:	>0.9999
			CON vs. VPA:	0.0011 **
			CON vs. RSV + VPA:	0.8282
			RSV vs. VPA:	0.0014 **
			RSV vs. RSV + VPA:	0.6359
			VPA vs. RSV + VPA:	<0.0001 ****
SOM Total aCC (IV/V)	CON: 33.25 ± 5.56 RSV: 30.50 ± 3.70 VPA: 21.00 ± 1.15 RSV + VPA: 31.25 ± 2.63	Interaction: F (1, 12) = 12.64 *p =* 0.0040 ** VPA: F (1, 12) = 9.888 *p =* 0.0085 ** RSV: F (1, 12) = 4.206 *p =* 0.0628 *	CON vs. RSV:	>0.9999
			CON vs. VPA:	0.0029 **
			CON vs. RSV + VPA:	>0.9999
			RSV vs. VPA:	0.0191 *
			RSV vs. RSV + VPA:	>0.9999
			VPA vs. RSV + VPA:	0.0113 *
SOM Ratio aCC (IV/V)	CON: 0.060 ± 0.010 RSV: 0.060 ± 0.008 VPA: 0.036 ± 0.007 RSV + VPA: 0.062 ± 0.005	Interaction: F (1, 12) = 15.49 *p =* 0.0020 ** VPA: F (1, 12) = 11.03 *p =* 0.0061 ** RSV: F (1, 12) = 13.84 *p =* 0.0029 **	CON vs. RSV:	0.9999
			CON vs. VPA:	0.0015 **
			CON vs. RSV + VPA:	0.9999
			RSV vs. VPA:	0.0019 **
			RSV vs. RSV + VPA:	0.9999
			VPA vs. RSV + VPA:	0.0009 ***
SOM Total Whole aCC	CON: 51.75 ± 4.20 RSV: 48.00 ± 4.00 VPA: 33.00 ± 1.15 RSV + VPA: 50.00 ± 4.32	Interaction: F (1, 12) = 31.75 *p =* 0.0001 *** VPA: F (1, 12) = 20.69 *p =* 0.0007 *** RSV: F (1, 12) = 12.94 *p =* 0.0037 **	CON vs. RSV:	>0.9999
			CON vs. VPA:	<0.0001 ****
			CON vs. RSV + VPA:	>0.9999
			RSV vs. VPA:	0.0005 ***
			RSV vs. RSV + VPA:	>0.9999
			VPA vs. RSV + VPA:	0.0002 ***
SOM Ratio Whole aCC	CON: 0.058 ± 0.006 RSV: 0.056 ± 0.007 VPA: 0.033 ± 0.005 RSV + VPA: 0.061 ± 0.005	Interaction: F (1, 12) = 23.64 *p =* 0.0004 *** VPA: F (1, 12) = 14.87 *p =* 0.0023 ** RSV: F (1, 12) = 21.35 *p =* 0.0006 ***	CON vs. RSV:	>0.9999
			CON vs. VPA:	0.0003 ***
			CON vs. RSV + VPA:	>0.9999
			RSV vs. VPA:	0.0004 ***
			RSV vs. RSV + VPA:	>0.9999
			VPA vs. RSV + VPA:	0.0001 ***
SOM Total PrL (II/III)	CON: 19.75 ± 8.25 RSV: 18.25 ± 1.55 VPA: 12.50 ± 0.70 RSV + VPA: 17.25 ± 1.90	Interaction: F (1, 12) = 2.825 *p =* 0.1186 VPA: F (1, 12) = 2.825 *p =* 0.1186 RSV: F (1, 12) = 0.2721 *p =* 0.6114	CON vs. RSV:	>0.9999
			CON vs. VPA:	0.2097
			CON vs. RSV + VPA:	>0.9999
			RSV vs. VPA:	0.8721
			RSV vs. RSV + VPA:	>0.9999
			VPA vs. RSV + VPA:	0.8721
SOM Ratio PrL (II/III)	CON: 0.053 ± 0.016 RSV: 0.047 ± 0.003 VPA: 0.032 ± 0.004 RSV + VPA: 0.049 ± 0.007	Interaction: F (1, 12) = 8.612 *p =* 0.0125 * VPA: F (1, 12) = 2.893 *p =* 0.1147 RSV: F (1, 12) = 3.103 *p =* 0.1036	CON vs. RSV:	>0.9999
			CON vs. VPA:	0.0396 *
			CON vs. RSV + VPA:	>0.9999
			RSV vs. VPA:	0.1841
			RSV vs. RSV + VPA:	>0.9999
			VPA vs. RSV + VPA:	0.0366 *
SOM Total PrL (IV/V)	CON: 37.00 ± 5.90 RSV: 34.25 ± 5.75 VPA: 21.25 ± 3.86 RSV + VPA: 29.25 ± 2.63	Interaction: F (1, 12) = 5.170 *p =* 0.0422 ** VPA: F (1, 12) = 19.26 *p =* 0.0009 *** RSV: F (1, 12) = 1.233 *p =* 0.2886	CON vs. RSV:	>0.9999
			CON vs. VPA:	0.0030 **
			CON vs. RSV + VPA:	0.2334
			RSV vs. VPA:	0.0129 *
			RSV vs. RSV + VPA:	0.9636
			VPA vs. RSV + VPA:	0.2037
SOM Ratio PrL (IV/V)	CON: 0.064 ± 0.008 RSV: 0.056 ± 0.008 VPA: 0.032 ± 0.007 RSV + VPA: 0.055 ± 0.005	Interaction: F (1, 12) = 18.82 *p =* 0.0010 VPA: F (1, 12) = 22.16 *p =* 0.0005 *** RSV: F (1, 12) = 4.211 *p =* 0.0626 #	CON vs. RSV:	0.7919
			CON vs. VPA:	0.0002 ***
			CON vs. RSV + VPA:	0.5095
			RSV vs. VPA:	0.0027**
			RSV vs. RSV + VPA:	>0.9999
			VPA vs. RSV + VPA:	0.0042 **
SOM Total Whole PrL	CON: 56.75 ± 12.55 RSV: 51.50 ± 7.25 VPA: 33.75 ± 3.40 RSV + VPA: 46.50 ± 2.00	Interaction: F (1, 12) = 5.739 *p =* 0.0338 * VPA: F (1, 12) = 13.89 *p =* 0.0029 ** RSV: F (1, 12) = 0.9963 *p =* 0.3379	CON vs. RSV:	>0.9999
			CON vs. VPA:	0.0059 **
			CON vs. RSV + VPA:	0.4661
			RSV vs. VPA:	0.0353 *
			RSV vs. RSV + VPA:	>0.9999
			VPA vs. RSV + VPA:	0.2012
SOM Ratio Whole PrL	CON: 0.060 ± 0.011 RSV: 0.053 ± 0.006 VPA: 0.031 ± 0.004 RSV + VPA: 0.054 ± 0.005	Interaction: F (1, 12) = 18.37 *p =* 0.0011 ** VPA: F (1, 12) = 15.21 *p =* 0.0021 ** RSV: F (1, 12) = 4.905 *p =* 0.0469 *	CON vs. RSV:	>0.9999
			CON vs. VPA:	0.0005 ***
			CON vs. RSV + VPA:	>0.9999
			RSV vs. VPA:	0.0059 **
			RSV vs. RSV + VPA:	>0.9999
			VPA vs. RSV + VPA:	0.0037 **
SOM Total IL (II/III)	CON: 15.50 ± 1.75 RSV: 17.00 ± 2.45 VPA: 11.50 ± 2.50 RSV + VPA: 15.25 ± 1.50	Interaction: F (1, 12) = 1,152 *p =* 0.3043 VPA: F (1, 12) = 7.521 *p =* 0.0178 * RSV: F (1, 12) = 6.270 *p =* 0.0277 *	CON vs. RSV:	>0.9999
			CON vs. VPA:	0.1163
			CON vs. RSV + VPA:	>0.9999
			RSV vs. VPA:	0.0179 *
			RSV vs. RSV + VPA:	>0.9999
			VPA vs. RSV + VPA:	0.1587
SOM Ratio IL (II/III)	CON: 0.045 ± 0.007 RSV: 0.048 ± 0.008 VPA: 0.032 ± 0.006 RSV + VPA: 0.048 ± 0.004	Interaction: F (1, 12) = 4.503 *p =* 0.0553 # VPA: F (1, 12) = 3.938 *p =* 0.0706 # RSV: F (1, 12) = 9.410 *p =* 0.0098 **	CON vs. RSV:	>0.9999
			CON vs. VPA:	0.0794 #
			CON vs. RSV + VPA:	>0.9999
			RSV vs. VPA:	0.0230 *
			RSV vs. RSV + VPA:	>0.9999
			VPA vs. RSV + VPA:	0.0193 *
SOM Total IL (IV/V)	CON: 34.50 ± 7.15 RSV: 33.75 ± 1.00 VPA: 21.75 ± 3.00 RSV + VPA: 33.00 ± 1.45	Interaction: F (1, 12) = 9.167 *p =* 0.0105 * VPA: F (1, 12) = 11,60 *p =* 0.0052 ** RSV: F (1, 12) = 7.019 *p =* 0.0212 *	CON vs. RSV:	>0.9999
			CON vs. VPA:	0.0040 **
			CON vs. RSV + VPA:	>0.9999
			RSV vs. VPA:	0.0064 **
			RSV vs. RSV + VPA:	>0.9999
			VPA vs. RSV + VPA:	0.0103*
SOM Ratio IL (IV/V)	CON: 0.058 ± 0.011 RSV: 0.052 ± 0.002 VPA: 0.032 ± 0.003 RSV + VPA: 0.059 ± 0.005	Interaction: F (1, 12) = 29.00 *p =* 0.0002 *** VPA: F (1, 12) = 9.617 *p =* 0.0092 ** RSV: F (1, 12) = 12.21 *p =* 0.0044 **	CON vs. RSV:	>0.9999
			CON vs. VPA:	0.0004 ***
			CON vs. RSV + VPA:	>0.9999
			RSV vs. VPA:	0.0033 **
			RSV vs. RSV + VPA:	0.7939
			VPA vs. RSV + VPA:	0.0002 ***
SOM Total Whole IL	CON: 50.00 ± 8.70 RSV: 50.75 ± 3.30 VPA: 33.25 ± 4.71 RSV + VPA: 48.25 ± 2.63	Interaction: F (1, 12) = 6.840 *p =* 0.0226 * VPA: F (1, 12) = 12.48 *p =* 0.0041 ** RSV: F (1, 12) = 8.356 *p =* 0.0136 *	CON vs. RSV:	>0.9999
			CON vs. VPA:	0.0057 **
			CON vs. RSV + VPA:	>0.9999
			RSV vs. VPA:	0.0041 **
			RSV vs. RSV + VPA:	>0.9999
			VPA vs. RSV + VPA:	0.0128 *
SOM Ratio Whole IL	CON: 0.053 ± 0.008 RSV: 0.051 ± 0.003 VPA: 0.032 ± 0.003 RSV + VPA: 0.055 ± 0.001	Interaction: F (1, 12) = 31.96 *p =* 0.0001 *** VPA: F (1, 12) = 12.93 *p =* 0.0037 ** RSV: F (1, 12) = 21.75 *p =* 0.0005 ***	CON vs. RSV:	>0.9999
			CON vs. VPA:	0.0002 ***
			CON vs. RSV + VPA:	>0.9999
			RSV vs. VPA:	0.0005 ***
			RSV vs. RSV + VPA:	>0.9999
			VPA vs. RSV + VPA:	<0.0001 ****

II/III, upper cortical layers; IV/V, deeper cortical layers; aCC, anterior cingulate cortex; IL, infralimbic cortex; mPFC, medial prefrontal cortex; PrL, prelimbic cortex; SD, standard deviation; SOM, somatostatin-positive interneuron. *p* < 0.05 was considered significant. * *p* < 0.05, ** *p* < 0.01, *** *p* < 0.001, **** *p* < 0.0001, # trend. Statistical analyses: two-way ANOVA parametric test followed by Bonferroni. N_CON_: 4. N_RSV_: 4. N_VPA_: 4. N_RSV + VPA_: 4.

## Data Availability

Data is contained within the article or Supplementary Material.

## Supplementary Materials

The following supporting information can be downloaded at https://www.mdpi.com/article/10.3390/ijms23084075/s1.
