# Supplementary material for: Resveratrol Prevents Cytoarchitectural and Interneuronal Alterations in the Valproic Acid Rat Model of Autism
Source: Int J Mol Sci. 2022 Apr 7;23(8):4075. doi: 10.3390/ijms23084075 (PMC9027778; doi:10.3390/ijms23084075)
Supplement: Supplementary file 1 [file ijms-23-04075-s001.zip › ijms-1662208-supplementary.pdf]

## Supplementary Table S1. Total Neurons distribution profile in HC

| | Mean ± SD | F (DFn. DFd); p Value | Pairwise comparisons | |
|---|---|---|---|---|
| **Total Neurons/Area**<br>**CA1** | CON: 0.0051±0.00087<br>RSV:0.0042±0.00052<br>VPA: 0.0041±0.00048<br>RSV+VPA:0.0045±0.00047 | Interaction:F (1, 12) = 4.036 p =0.0676#<br>VPA: F (1, 12) = 1.210 p =0.2929<br>RSV: F (1, 12) = 0.6974 p =0.4200 | CON vs RSV: | 0.4040 |
| | | | CON vs VPA: | 0.2896 |
| | | | CON vs RSV+VPA: | > 0.9999 |
| | | | RSV vs VPA: | > 0.9999 |
| | | | RSV vs RSV+VPA: | > 0.9999 |
| | | | VPA vs RSV+VPA: | > 0.9999 |
| **Total Neurons/Area**<br>**CA2** | CON: 0.0030±0.00013<br>RSV:0.0027±0.000037<br>VPA: 0.0026±0.00027<br>RSV+VPA:0.0031±0.00031 | Interaction:F (1, 12) = 12.45 p =0.0042**<br>VPA: F (1, 12) = 0.007008 p =0.9347<br>RSV: F (1, 12) = 1.116 p =0.3117 | CON vs RSV: | 0.6355 |
| | | | CON vs VPA: | 0.1516 |
| | | | CON vs RSV+VPA: | > 0.9999 |
| | | | RSV vs VPA: | > 0.9999 |
| | | | RSV vs RSV+VPA: | 0.1883 |
| | | | VPA vs RSV+VPA: | 0.0424* |
| **Total Neurons/Area**<br>**CA3** | CON: 0.0029±0.00058<br>RSV: 0.0027±0.00029<br>VPA: 0.0028±0.00087<br>RSV+VPA:0.0032±0.0034 | Interaction:F (1, 12) = 1.494 p =0.2450<br>VPA: F (1, 12) = 0.9729 p =0.3434<br>RSV: F (1, 12) = 0.6828 p =0.4247 | CON vs RSV: | > 0.9999 |
| | | | CON vs VPA: | > 0.9999 |
| | | | CON vs RSV+VPA: | > 0.9999 |
| | | | RSV vs VPA: | > 0.9999 |
| | | | RSV vs RSV+VPA: | 0.8657 |
| | | | VPA vs RSV+VPA: | > 0.9999 |

**HC**: hippocampus; **SD**: standard deviation. p <0.05 was considered significant. *p<0.05. **p<0.01, #trend. Statistical analyses: two-way ANOVA parametric test followed by Bonferroni. $N_{CON}$: 4. $N_{RSV}$: 4. $N_{VPA}$: 4. $N_{RSV+VPA}$: 4.

## Supplementary Table S2. Distribution profile of PV neurons in the HC

| | Mean ± SD | F (DFn. DFd); p Value | Pairwise comparisons | |
|---|---|---|---|---|
| **PV<br>/Area<br><br>CA1** | CON: 0.00022±0.000023<br>RSV:0.00021±0.000030<br>VPA:0.00023±0.000040<br>RSV+VPA:0.00020±0.000030 | Interaction: F (1, 12) = 0.322 p =0.580<br>VPA: F (1, 12) = 0.03768 p =0.8493<br>RSV: F (1, 12) = 1.685 p =0.2186 | CON vs RSV: | > 0.9999 |
| | | | CON vs VPA: | > 0.9999 |
| | | | CON vs RSV+VPA: | > 0.9999 |
| | | | RSV vs VPA: | > 0.9999 |
| | | | RSV vs RSV+VPA: | > 0.9999 |
| | | | VPA vs RSV+VPA: | > 0.9999 |
| **PV Ratio<br><br>CA1** | CON: 0.054±0.009<br>RSV:0.047±0.008<br>VPA: 0.057±0.005<br>RSV+VPA:0.050±0.011 | Interaction:F (1, 12) = 0.0014 p =0.970<br>VPA: F (1, 12) = 0.4375 p =0.5208<br>RSV: F (1, 12) = 3.089 p =0.1043 | CON vs RSV: | > 0.9999 |
| | | | CON vs VPA: | > 0.9999 |
| | | | CON vs RSV+VPA: | > 0.9999 |
| | | | RSV vs VPA: | 0.6772 |
| | | | RSV vs RSV+VPA: | > 0.9999 |
| | | | VPA vs RSV+VPA: | > 0.9999 |
| **PV/Area<br><br>CA2** | CON: 0.00016±0.000036<br>RSV:0.00014±0.000035<br>VPA: 0.00016±0.000021<br>RSV+VPA:0.00012±0.000024 | Interaction: F (1, 12) = 1.367 p =0.265<br>VPA: F (1, 12) = 0.7516 p =0.4030<br>RSV: F (1, 12) = 3.561 p =0.0836# | CON vs RSV: | > 0.9999 |
| | | | CON vs VPA: | > 0.9999 |
| | | | CON vs RSV+VPA: | 0.4516 |
| | | | RSV vs VPA: | > 0.9999 |
| | | | RSV vs RSV+VPA: | > 0.9999 |
| | | | VPA vs RSV+VPA: | 0.3096 |
| **PV Ratio<br><br>CA2** | CON: 0.044±0.008<br>RSV:0.057±0.005<br>VPA: 0.050±0.013<br>RSV+VPA: 0.043±0.011 | Interaction:F (1, 12) = 4.109 p =0.0655#<br>VPA: F (1, 12) = 0.6387 p =0.4397<br>RSV: F (1, 12) = 0.3637 p =0.5577 | CON vs RSV: | 0.05256 |
| | | | CON vs VPA: | > 0.9999 |
| | | | CON vs RSV+VPA: | > 0.9999 |
| | | | RSV vs VPA: | > 0.9999 |
| | | | RSV vs RSV+VPA: | 0.4130 |
| | | | VPA vs RSV+VPA: | > 0.9999 |
| **PV /Area<br><br>CA3** | CON:0.00023±0.000043<br>RSV:0.00018±0.000017<br>VPA:0.00023± 0.000040<br>RSV+VPA: 0.00016±0.000019 | Interaction: F (1, 12) = 0.975 p =0.342<br>VPA: F (1, 12) = 0.6635 p =0.4312<br>RSV: F (1, 12) = 16.27 p =0.0017** | CON vs RSV: | 0.3139 |
| | | | CON vs VPA: | > 0.9999 |
| | | | CON vs RSV+VPA: | 0.0300* |
| | | | RSV vs VPA: | 0.2519 |
| | | | RSV vs RSV+VPA: | > 0.9999 |
| | | | VPA vs RSV+VPA: | 0.0240* |
| **PV Ratio<br><br>CA3** | CON:0.072±0.013<br>RSV:0.063±0.004<br>VPA:0.059±0.013<br>RSV+VPA: 0.052±0.011 | Interaction:F (1, 12) = 0.0289 p =0.867<br>VPA: F (1, 12) = 4.742 p =0.0501#<br>RSV: F (1, 12) = 2.184 p =0.1652 | CON vs RSV: | > 0.9999 |
| | | | CON vs VPA: | 0.7366 |
| | | | CON vs RSV+VPA: | 0.1433 |
| | | | RSV vs VPA: | > 0.9999 |
| | | | RSV vs RSV+VPA: | > 0.9999 |
| | | | VPA vs RSV+VPA: | > 0.9999 |

**PV**: parvalbumin-positive interneuron; **HC**: hippocampus; **SD**: standard deviation. p <0.05 was considered significant. *p<0.05. **p<0.01, #trend. Statistical analyses: two-way ANOVA parametric test followed by Bonferroni. $N_{CON}$: 4. $N_{RSV}$: 4. $N_{VPA}$: 4. $N_{RSV+VPA}$: 4.

## Supplementary Table S3. Distribution profile of CB neurons in the HC

| | Mean ± SD | F (DFn. DFd); p Value | Pairwise comparisons | |
|---|---|---|---|---|
| **CB /Area CA1** | CON: 0.000072±0.000014<br>RSV: 0.000097±0.000019<br>VPA: 0.000044±0.000028<br>RSV+VPA: 0.000045±0.000015 | Interaction:F (1, 11) = 1.151 p =0.3062<br>VPA: F (1, 11) = 14.32 p =0.0030**<br>RSV: F (1, 11) = 1.421 p =0.2583 | CON vs RSV: | 0.7420 |
| | | | CON vs VPA: | 0.4284 |
| | | | CON vs RSV+VPA: | 0.6306 |
| | | | RSV vs VPA: | 0.0224* |
| | | | RSV vs RSV+VPA: | 0.0418* |
| | | | VPA vs RSV+VPA: | > 0.9999 |
| **CB Ratio CA1** | CON: 0.017±0.003<br>RSV: 0.024±0.003<br>VPA: 0.015±0.010<br>RSV+VPA: 0.016±0.008 | Interaction:F (1, 12) = 0.782 p =0.3938<br>VPA: F (1, 12) = 1.679 p =0.2194<br>RSV: F (1, 12) = 1.258 p =0.2839 | CON vs RSV: | 0.9263 |
| | | | CON vs VPA: | > 0.9999 |
| | | | CON vs RSV+VPA: | > 0.9999 |
| | | | RSV vs VPA: | 0.6471 |
| | | | RSV vs RSV+VPA: | > 0.9999 |
| | | | VPA vs RSV+VPA: | > 0.9999 |
| **CB /Area CA2** | CON: 0.000035±0.0000053<br>RSV:0.000050±0.000022<br>VPA:0.000010±0.0000021<br>RSV+VPA:0.000026±0.000022 | Interaction:F (1, 11) = 0.0014 p =0.971<br>VPA: F (1, 11) = 9.038 p =0.0119*<br>RSV: F (1, 11) = 3.388 p =0.0928# | CON vs RSV: | > 0.9999 |
| | | | CON vs VPA: | 0.2801 |
| | | | CON vs RSV+VPA: | > 0.9999 |
| | | | RSV vs VPA: | 0.0265* |
| | | | RSV vs RSV+VPA: | 0.4084 |
| | | | VPA vs RSV+VPA: | > 0.9999 |
| **CB Ratio CA2** | CON: 0.015±0.004<br>RSV:0.018±0.006<br>VPA: 0.006±0.003<br>RSV+VPA: 0.009±0.006 | Interaction:F (1, 11) = 0.00547 p =0.942<br>VPA: F (1, 11) = 10.50 p =0.0079**<br>RSV: F (1, 11) = 1.671 p =0.2226 | CON vs RSV: | > 0.9999 |
| | | | CON vs VPA: | 0.1972 |
| | | | CON vs RSV+VPA: | > 0.9999 |
| | | | RSV vs VPA: | 0.0398* |
| | | | RSV vs RSV+VPA: | 0.3233 |
| | | | VPA vs RSV+VPA: | > 0.9999 |
| **CB /Area CA3** | CON:0.000069±0.000021<br>RSV: 0.000068±0.000014<br>VPA: 0.000044±0.000019<br>RSV+VPA: 0.000030±0.000013 | Interaction:F (1, 10) = 0.625 p =0.4475<br>VPA: F (1, 10) = 11.52 p =0.0068**<br>RSV: F (1, 10) = 0.7661 p =0.4020 | CON vs RSV: | > 0.9999 |
| | | | CON vs VPA: | 0.5725 |
| | | | CON vs RSV+VPA: | 0.1082 |
| | | | RSV vs VPA: | 0.4995 |
| | | | RSV vs RSV+VPA: | 0.0858# |
| | | | VPA vs RSV+VPA: | > 0.9999 |
| **CB Ratio CA3** | CON: 0.022±0.005<br>RSV: 0.022±0.006<br>VPA: 0.013±0.004<br>RSV+VPA: 0.09±0.003 | Interaction: F (1, 10) = 0.679 p =0.429<br>VPA: F (1, 10) = 19.20 p =0.0014**<br>RSV: F (1, 10) = 0.8092 p =0.3895 | CON vs RSV: | > 0.9999 |
| | | | CON vs VPA: | 0.1836 |
| | | | CON vs RSV+VPA: | 0.0347* |
| | | | RSV vs VPA: | 0.1434 |
| | | | RSV vs RSV+VPA: | 0.0254* |
| | | | VPA vs RSV+VPA: | > 0.9999 |

**CB**: calbindin-positive interneuron; **HC**: hippocampus; **SD**: standard deviation. p <0.05 was considered significant. *p<0.05. **p<0.01, #trend. Statistical analyses: two-way ANOVA parametric test followed by Bonferroni. $N_{CON}$: 4. $N_{RSV}$: 4. $N_{VPA}$: 4. $N_{RSV+VPA}$: 3.

## Supplementary Table S4. Distribution profile of SOM neurons in the HC

| | Mean ± SD | F (DFn. DFd); p Value | Pairwise comparisons | |
|---|---|---|---|---|
| **SOM /Area**<br>**CA1** | CON: 0.00017±0.000031<br>RSV:0.00015±0.000026<br>VPA: 0.000168±0.000057<br>RSV+VPA: 0.00021±0.000048 | Interaction:F (1, 12) = 2.670 p =0.1282<br>VPA: F (1, 12) = 2.302 p =0.1551<br>RSV: F (1, 12) = 0.8441 p =0.3763 | CON vs RSV: | > 0.9999 |
| | | | CON vs VPA: | > 0.9999 |
| | | | CON vs RSV+VPA: | 0.6637 |
| | | | RSV vs VPA: | > 0.9999 |
| | | | RSV vs RSV+VPA: | 0.2745 |
| | | | VPA vs RSV+VPA: | 0.5772 |
| **SOM Ratio**<br>**CA1** | CON: 0.034±0.0098<br>RSV: 0.039±0.0047<br>VPA: 0.044±0.0016<br>RSV+VPA: 0.045±0.0052 | Interaction:F (1, 12) = 0.0685 p =0.797<br>VPA:F (1, 12) = 2.754 p =0.1229<br>RSV: F (1, 12) = 0.4506 p =0.5148 | CON vs RSV: | > 0.9999 |
| | | | CON vs VPA: | > 0.9999 |
| | | | CON vs RSV+VPA: | 0.7516 |
| | | | RSV vs VPA: | > 0.9999 |
| | | | RSV vs RSV+VPA: | > 0.9999 |
| | | | VPA vs RSV+VPA: | > 0.9999 |
| **SOM/Area**<br>**CA2** | CON: 0.00011±0.000017<br>RSV: 0.00010±0.000018<br>VPA: 0.000084±0.000013<br>RSV+VPA:0.000088±0.00001 | Interaction:F (1, 12) = 1.030 p =0.3301<br>VPA: F (1, 12) = 8.942 p =0.0113*<br>RSV: F (1, 12) = 0.2051 p =0.6587 | CON vs RSV: | > 0.9999 |
| | | | CON vs VPA: | 0.0907# |
| | | | CON vs RSV+VPA: | 0.1888 |
| | | | RSV vs VPA: | 0.5879 |
| | | | RSV vs RSV+VPA: | > 0.9999 |
| | | | VPA vs RSV+VPA: | > 0.9999 |
| **SOM Ratio**<br>**CA2** | CON: 0.038±0.0054<br>RSV: 0.043±0.0059<br>VPA: 0.032±0.0066<br>RSV+VPA:0.031±0.0031 | Interaction:F (1, 12) = 1.866 p =0.1970<br>VPA: F (1, 12) = 11.62 p =0.0052**<br>RSV: F (1, 12) = 0.4153 p =0.531 | CON vs RSV: | > 0.9999 |
| | | | CON vs VPA: | > 0.9999 |
| | | | CON vs RSV+VPA: | 0.4460 |
| | | | RSV vs VPA: | 0.0852# |
| | | | RSV vs RSV+VPA: | 0.0330* |
| | | | VPA vs RSV+VPA: | > 0.9999 |
| **SOM /Area**<br>**CA3** | CON: 0.00010±0.000020<br>RSV: 0.00014±0.000016<br>VPA: 0.00010±0.000035<br>RSV+VPA: 0.00014±0.000029 | Interaction:F (1, 12) = 0.0885 p =0.771<br>VPA: F (1, 12) = 0.04458 p =0.8363<br>RSV: F (1, 12) = 8.007 p =0.0152* | CON vs RSV: | 0.5916 |
| | | | CON vs VPA: | > 0.9999 |
| | | | CON vs RSV+VPA: | 0.3157 |
| | | | RSV vs VPA: | 0.5330 |
| | | | RSV vs RSV+VPA: | > 0.9999 |
| | | | VPA vs RSV+VPA: | 0.2830 |
| **SOM Ratio**<br>**CA3** | CON: 0.037±0.012<br>RSV: 0.050±0.006<br>VPA: 0.038±0.008<br>RSV+VPA: 0.044±0.013 | Interaction:F (1, 12) = 0.498 p =0.4935<br>VPA: F (1, 12) = 0.3335 p =0.5743<br>RSV: F (1, 12) = 3.713 p =0.0780# | CON vs RSV: | 0.5237 |
| | | | CON vs VPA: | > 0.9999 |
| | | | CON vs RSV+VPA: | > 0.9999 |
| | | | RSV vs VPA: | 0.6118 |
| | | | RSV vs RSV+VPA: | > 0.9999 |
| | | | VPA vs RSV+VPA: | > 0.9999 |

**SOM**: somatostatin-positive interneuron; **HC**: hippocampus; **SD**: standard deviation. p <0.05 was considered significant. *p<0.05. **p<0.01, #trend. Statistical analyses: two-way ANOVA parametric test followed by Bonferroni. $N_{CON}$: 4. $N_{RSV}$: 4. $N_{VPA}$: 4. $N_{RSV+VPA}$: 4.

## Supplementary Table S5 – Information on key immunofluorescence and western blotting reagents

| Immunofluorescence | | | |
|---|---|---|---|
| **Reagent** | **Supplier** | **Code** | **Dilution and Time** |
| Anti-NeuN (Mouse) | Merck | MAB3771 | 1:500 in PBS-Triton 0.1% overnight at 4ºC |
| Anti-NeuN | Abcam | Ab177487 | 1:500 in PBS-Triton 0.1% overnight at 4ºC |
| Anti-Parvalbumin | Abcam | Ab64555 | 1:500 in PBS-Triton 0.1% overnight at 4ºC |
| Anti-Calbindin | Abcam | Ab82812 | 1:500 in PBS-Triton 0.1% overnight at 4ºC |
| Anti-Somatostatin | Sigma-Aldrich | SAB4502861 | 1:500 in PBS-Triton 0.1% overnight at 4ºC |
| Alexa Fluor 488 Goat Anti-Mouse IgG (H+L) | Molecular Probes | Ab11029 | 1:2000 in PBS-Triton 0.1% in room temperature for 1h |
| Alexa Fluor 594 Goat Anti-Rabbit IgG (H+L) | Molecular Probes | Ab150080 | 1:2000* in PBS-Triton 0.1% in room temperature for 1h |
| DAPI Nucleic Acid Stain | Invitrogen | MP01306 | 30 uL per slice |
| Mounting Medium Fluorshield | Sigma-Aldrich | F6182-20ML | 30 uL per slice |

* In $SOM^+$ assay, the concentration was 1:1000

| Western blotting | | | |
|---|---|---|---|
| **Reagent** | **Supplier** | **Code** | **Dilution** |
| $GABA_A$ (goat) | Santa Cruz Biotechnology | sc-31405 | 1:250 in 5% BSA-0.1%TTBS |
| $GABA_B$ (rabbit) | Santa Cruz Biotechnology | sc-14006 | 1:500 in 5% BSA-0.1%TTBS |
| Gephyrin (rabbit) | Abcam | ab32206 | 1:500 in 5% BSA-0.1%TTBS |
| Neuroligin-2 (rabbit) | Abcam | ab36602 | 1:500 in 5% milk -0.1%TTBS |
| PSD-95 (rabbit) | Abcam | ab18258 | 1:500 in 5% BSA-0.1%TTBS |
| Synaptophysin (mouse) | SIGMA Aldrich | S5768 | 1:1000 in 5% BSA-0.1%TTBS |
| Anti-β-actin (mouse) | SIGMA Aldrich | A1978 | 1:3000 in TTBS |
| IgG-HRP (goat) | Santa Cruz Biotechnology | sc-2354 | 1:1000 in 5% BSA-0.1%TTBS |
| IgG-HRP (mouse) | Santa Cruz Biotechnology | sc-2314 | 1:1000 in 5% milk-0.1%TTBS |
| IgG-HRP (rabbit) | Santa Cruz Biotechnology | sc-2004 | 1:1000 in 0.1%TTBS |

| Common reagents | |
|---|---|
| PBS | 0.1 M pH 7.4 Phosphate-buffered saline |
| SDS 10% | Sodium dodecyl sulfate |
| EDTA 100 mM | Ethylenediamine tetraacetic acid |
| TRIS/HCl buffer 500 mM pH 8 | Tris(hydroxymethyl)aminomethane and chloridric acid |
| TBS | TRIS buffer |
| TTBS | 0.1%Tween20-TBS |

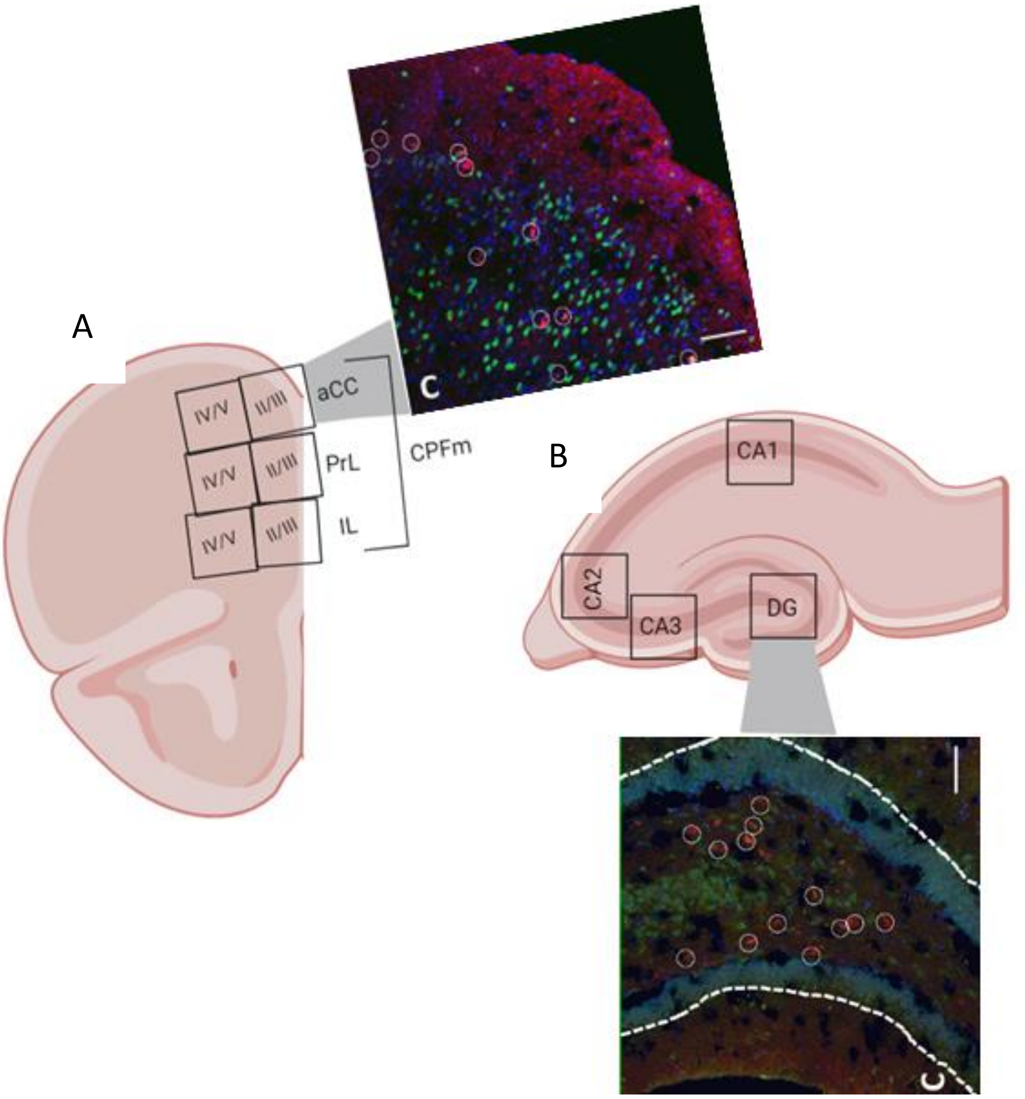


**Figure S1.** Illustrative scheme of the regions analyzed in this study. **A**) Representation of the mPFC and the three subregions evaluated (aCC, PrL, and IL), each one subdivided according to the cortical layers: upper layers (II/III) and deeper layers (IV/V). The total number of neurons, the number of each interneuron (PV+, CB+, and SOM+), and the ratio (interneuron/total neurons) were evaluated in both subdivisions of cortical layers in the three subregions. The amount observed in the deeper + the upper layers of a subregion was added to calculate the total for the whole subregion. Data for the whole mPFC was calculated as the sum of the amount observed in alle subregions. **B**) Representation of the hippocampus and the four evaluated subregions (DG, CA1, CA2, and CA3). In each of them, the total number of neurons, the number of each interneuron (PV+, CB+, and SOM+), and the ratio (interneuron/total neurons) were evaluated. **C**) Illustrative image. aCC, anterior cingulate cortex; CA, cornu ammonis; CB, calbindin-neurons; DG, dentate gyrus; IL, infralimbic cortex; mPFC, medial frontal cortex; PrL, prelimbic cortex; PV, parvalbumin-neurons; SOM, somatostatin-neurons.

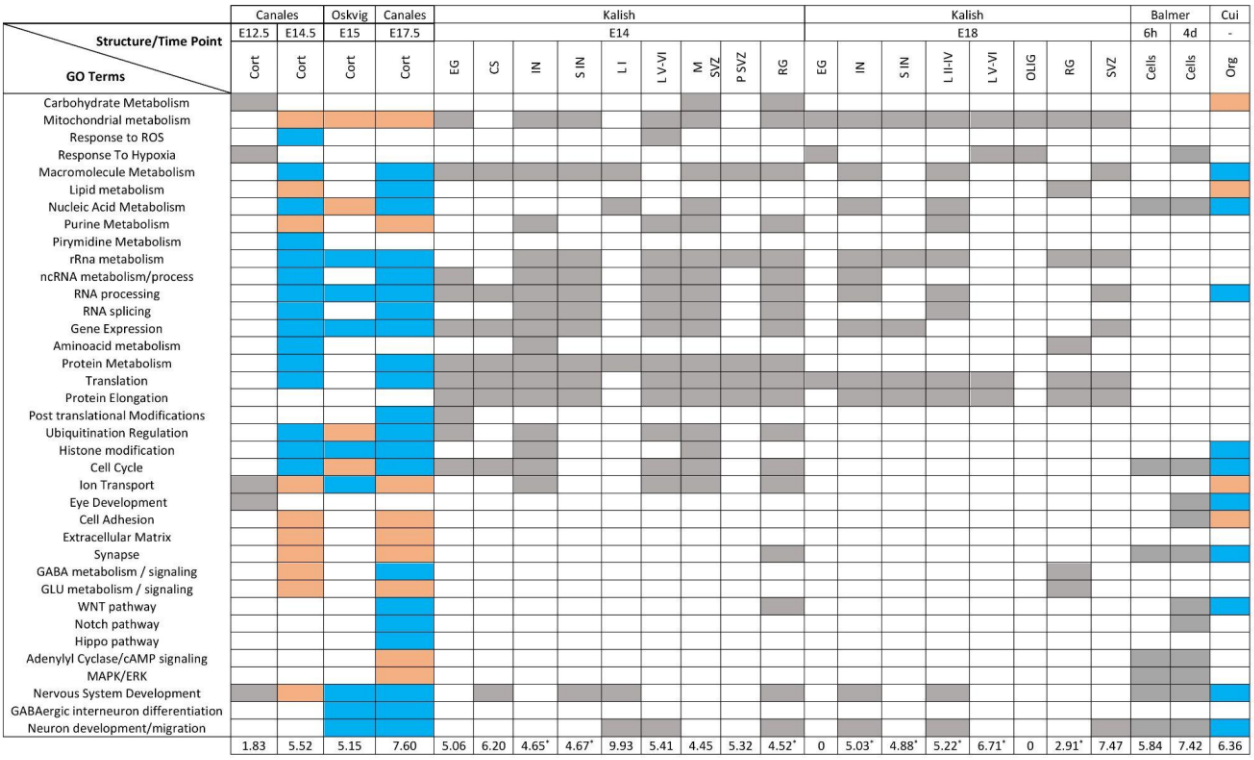


**Figure S2.** Compilation of the databases from ASD or associated models analyzed in this study. The five databases (described in Table 1) are organized according to the time-point of embryo development (where applicable) with the most relevant pathways listed in the left column. Gray squares represent pathways associated with the DGEs in general; Orange squares represent pathways specifically associated with upregulated DGEs; and blue squares represent pathways specifically associated with downregulated DGEs. The numbers in the bottom line represent the percentage of orthologs identified in the SFARI database, *indicate that a mean was obtained from all of the cells associated with the specific region. Cort.: cortex; CS: cortical subplate; DGE: differentially expressed genes; GE: ganglionic eminence; IN: interneuron; L: layer; OLIG: oligodendrocyte; Org: organoid; RG: radial glia; S IN: striatal interneuron; SVZ: subventricular zone.

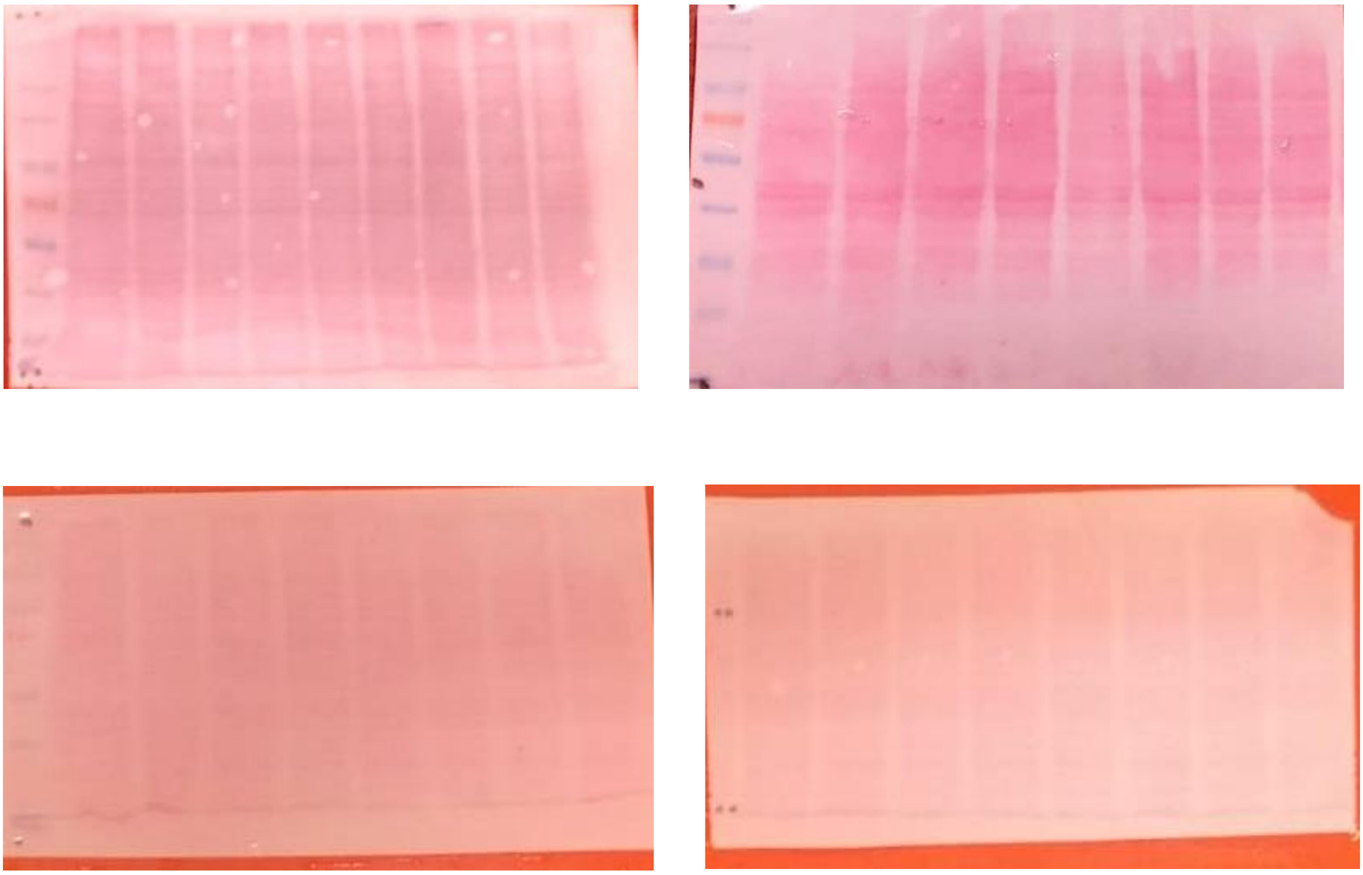

**Supplementary Figure S3. Ponceau staining of the nitrocellulose membranes – mPFC**

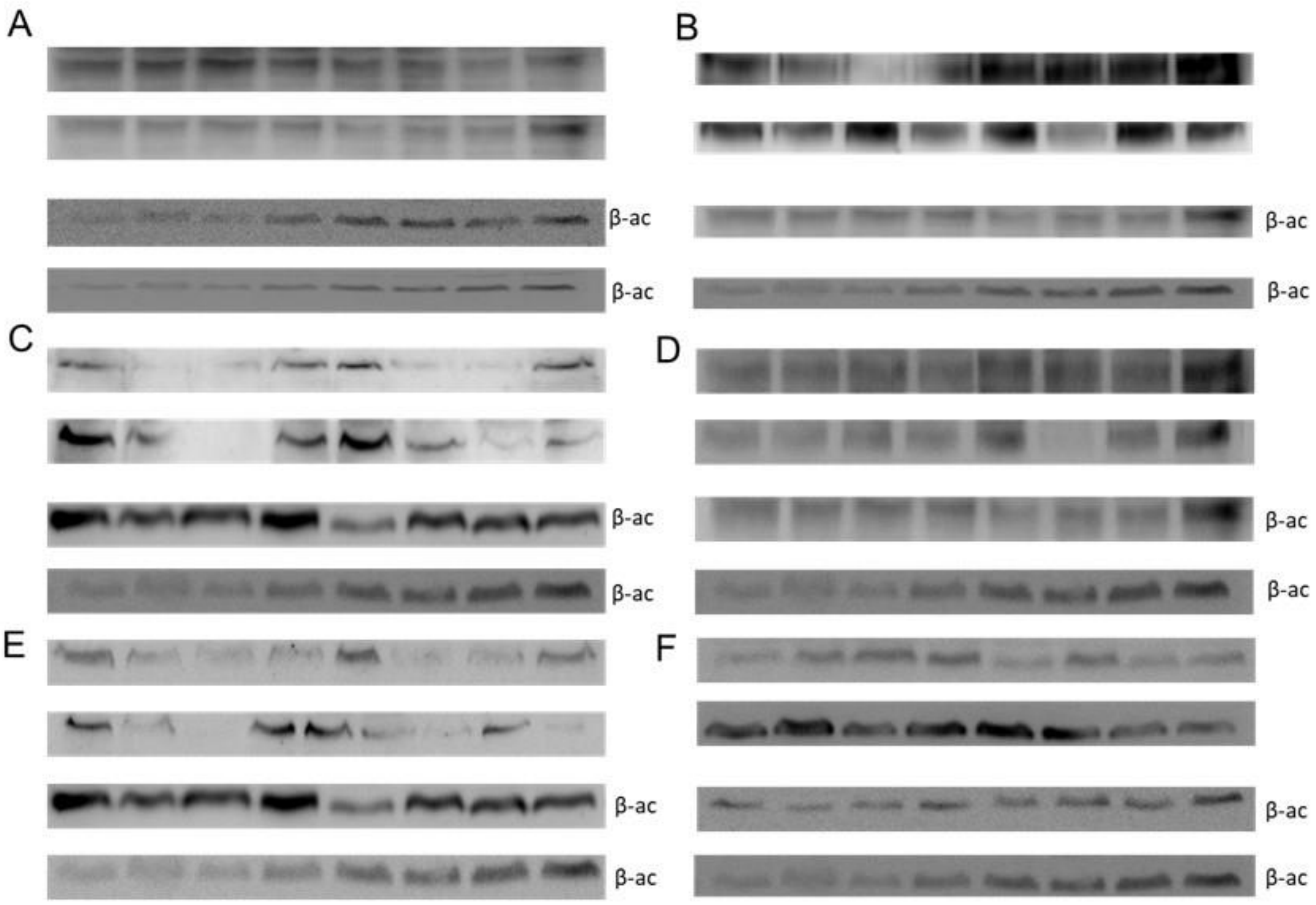


**Supplementary Figure S4**. **Nitrocellulose membranes mPFC**. A) $GABA_A$; B) $GABA_B$; C) Gephyrin; D) PSD-95; E) Neurolygin-2; F) Synaptophysin. In the image, β-ac stands for β-actin.

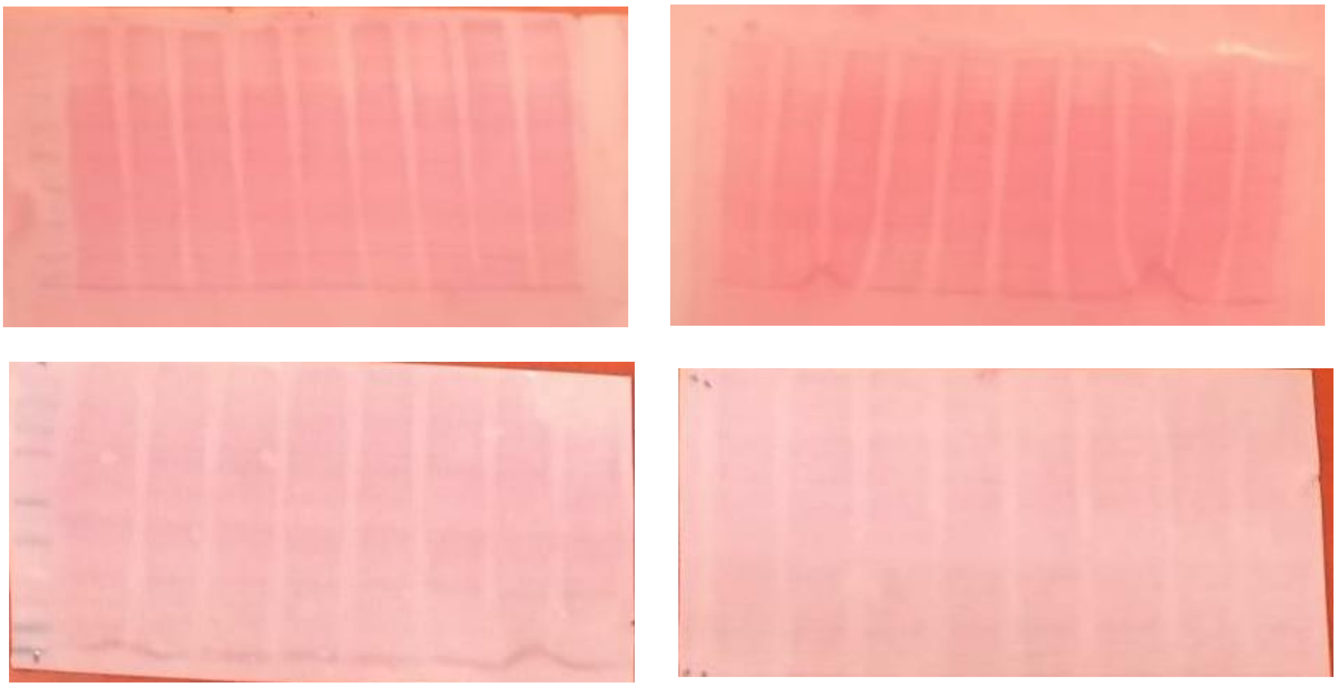

**Supplementary Figure S5**. **Ponceau staining of the nitrocellulose membranes – HC**

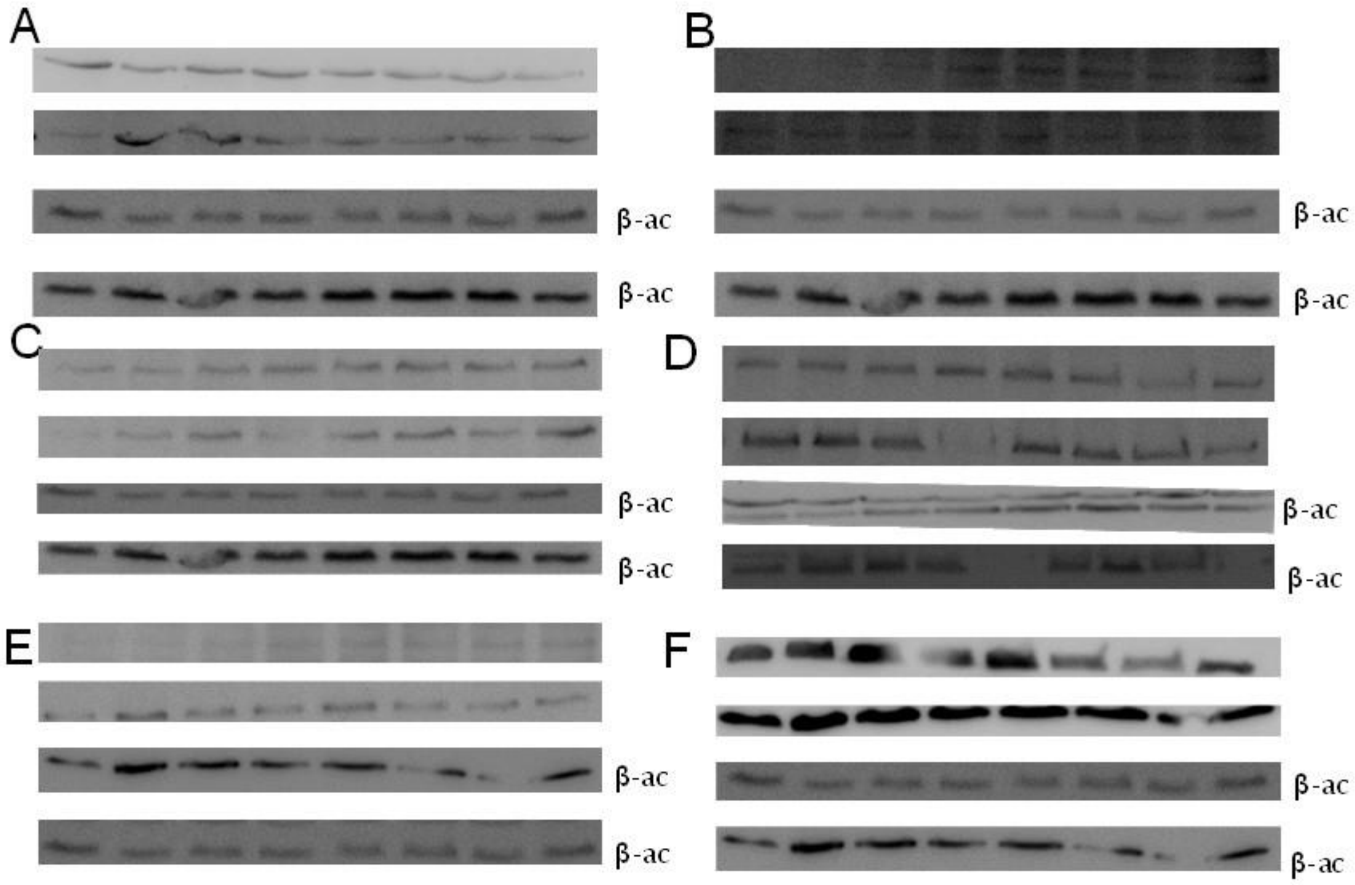


**Supplementary Figure S6**. **Nitrocellulose membranes HC**. A) $GABA_A$; B) $GABA_B$; C) Gephyrin; D) PSD-95; E) Neurolygin-2; F) Synaptophysin. In the image, β-ac stands for β-actin.
